# Consequences of the perivascular niche remodeling for tumoricidal T-cell trafficking into metastasis of ovarian cancer

**DOI:** 10.1093/immhor/vlaf084

**Published:** 2026-02-12

**Authors:** Marta Winkler, Nemi Malhotra, Anna Mistarz, Sophie Wang, Alan Hutson, Andrea Gambotto, Scott I Abrams, Prashant K Singh, Song Liu, Kunle O Odunsi, Jianmin Wang, Danuta Kozbor

**Affiliations:** Department of Immunology, Roswell Park Comprehensive Cancer Center, Buffalo, NY, United States; Department of Immunology, Roswell Park Comprehensive Cancer Center, Buffalo, NY, United States; Department of Immunology, Roswell Park Comprehensive Cancer Center, Buffalo, NY, United States; Department of Biostatistics and Bioinformatics, Roswell Park Comprehensive Cancer Center, Buffalo, NY, United States; Department of Biostatistics and Bioinformatics, Roswell Park Comprehensive Cancer Center, Buffalo, NY, United States; Department of Surgery, University of Pittsburgh, Pittsburgh, PA, United States; Department of Immunology, Roswell Park Comprehensive Cancer Center, Buffalo, NY, United States; Department of Cancer Genetics and Genomics, Roswell Park Comprehensive Cancer Center, Buffalo, NY, United States; Department of Biostatistics and Bioinformatics, Roswell Park Comprehensive Cancer Center, Buffalo, NY, United States; Department of Obstetrics and Gynecology, University of Chicago Comprehensive Cancer Center, Chicago, IL, United States; Department of Biostatistics and Bioinformatics, Roswell Park Comprehensive Cancer Center, Buffalo, NY, United States; Department of Immunology, Roswell Park Comprehensive Cancer Center, Buffalo, NY, United States

**Keywords:** CXCR4 antagonist, immunotherapy, oncolytic vaccinia virus, ovarian cancer, tumor vasculature

## Abstract

Aberrant angiogenesis in ovarian cancer (OC), driven by excessive vascular endothelial growth factor (VEGF) and other proangiogenic mediators, gives rise to structurally and functionally abnormal tumor vasculature that hinders effective T-cell infiltration. To overcome these barriers, we investigated how modulation of the perivascular niche influences antitumor T-cell trafficking and function in OC. T cells expressing a rearranged TCR transgene specific for SV40 T antigen (TAG) were adoptively transferred into TAG^+^ MOVCAR 5009 ovarian tumor–bearing SCID mice or syngeneic *TgMISIIR-TAg-Low* transgenic mice, which express TAG as a self-antigen in the fallopian tube epithelium. Transfers were performed either alone or following treatment with an oncolytic vaccinia virus expressing a CXCR4 antagonist (OV-CXCR4-A) or a control virus (OV-Fc). Compared with OV-Fc, OV-CXCR4-A treatment remodeled the tumor vasculature, inhibited recruitment of VEGF-producing myeloid-derived suppressor cells, and disrupted the proangiogenic microenvironment. These changes enhanced infiltration of adoptively transferred TCR_TAG_ T cells within the perivascular niche, correlating with improved antitumor activity and survival. Collectively, our findings demonstrate that CXCR4 blockade–mediated reprogramming of the perivascular tumor microenvironment promotes effective T-cell trafficking and function, providing a mechanistic rationale for combining oncolytic virotherapy with adoptive cell transfer in OC.

## Introduction

The limited therapeutic benefit of immune checkpoint blockades (ICBs)^1^^,^^2^ in ovarian cancer (OC) patients underscores the need for novel approaches to improve ICB efficacy that take into account a better understanding of how tumoricidal T cells traffic across tumor vascular checkpoints.^3^^,^^4^ Overexpression of tumor- or stromal-derived proangiogenic molecules, such as vascular endothelial growth factor (VEGF), results in the formation of immature, tortuous, and hyperpermeable aberrant blood vessels, which are inefficient at nutrient delivery and T-cell trafficking.^5^

Given the intricate relationship between the tumor vasculature and the tumor microenvironment (TME), combining antiangiogenic therapy with immunotherapy has been explored in various preclinical models to overcome barriers to impaired T-cell trafficking into the TME.^3^ However, a deeper understanding of the pathways regulating T-cell infiltration and activity in OC is needed to identify strategies that could be exploited therapeutically to enhance the delivery of antitumor T cells to ovarian tumors. Among various immunotherapeutic approaches, oncolytic viruses that target the tumor vasculature by infecting proliferating tumor vascular endothelial cells (ECs)^6^ and inducing immunogenic cell death (ICD)^7^^,^^8^ represent a promising multifaceted class of tumor therapeutics.^9^ Our previous studies demonstrated that treatment of syngeneic mice bearing ovarian or breast tumors with an oncolytic vaccinia virus (OV) expressing a CXCR4 antagonist (OV-CXCR4-A) significantly reduced tumor burden. This effect was mediated through the induction of ICD-mediated antitumor immune responses, attenuation of the immunosuppressive network, and inhibition of vasculogenesis and angiogenesis.^10–12^ However, it remains unclear to what extent OV-CXCR4-A–induced alterations in the tumor vasculature versus modulation of the perivascular niche within the TME contribute to enhanced CD8^+^ T-cell infiltration and the associated survival benefit.

In this study, we investigated how modulation of the perivascular niche influences tumor-specific T-cell trafficking and function in ovarian tumor–bearing mice. Specifically, we analyzed the crosstalk between adoptively transferred T cells expressing a T-cell receptor (TCR) transgene specific for the *H2-D^b^*-restricted SV40 large T antigen (TAG)_(206-215)_ epitope I and the perivascular TME of TAG^+^ MOVCAR 5009 omental tumors^13^ in SCID mice, and subsequently in syngeneic *TgMISIIR-TAg-Low* transgenic mice expressing TAG as a self-antigen in the fallopian tube epithelium.^11^^,^^14^ To assess how virally delivered co-stimulatory danger signals influence the acquisition of effector function by tumor-infiltrating lymphocytes upon migration into the tumor bed, mice were treated with an oncolytic vaccinia virus expressing a CXCR4 antagonist (OV-CXCR4-A) or a control virus expressing the Fc region of IgG2a (OV-Fc) prior to intravenous adoptive cell transfer (ACT). CXCR4 blockade-mediated remodeling of the perivascular niche altered tumor vasculature, enhanced T-cell infiltration, and improved therapeutic efficacy. Moreover, the extent of adoptively transferred TCR_TAG_ T-cell accumulation within the perivascular niche correlated with their activation state, which was influenced by the treatment-induced immunosuppressive network, particularly in infiltrating myeloid cell populations.

## Materials and methods

### Mice and cells

Six- to 8-week-old female B6.Cg-Tg(TcraY1, TcrbY1)416Tev/J mice, expressing a rearranged TCR transgene specific for the *H2-D^b^*-restricted SV40 large tumor antigen (TAG)_206-215_ epitope I (SAINNYAQKL), were obtained from The Jackson Laboratory (Bar Harbor, ME, USA) and housed in the Comparative Oncology Shared Resource (COSR) at Roswell Park Comprehensive Cancer Center (RPCCC; Buffalo, NY, USA). Six-week-old female C.B-Igh-1b-Icr-Tac-Prkdc SCID/Ros (SCID) mice were obtained from the COSR at RPCCC. Six-week-old female C57BL/6 *TgMISIIR-TAg-Low* transgenic mice (referred to as *TgMISIIR-TAg-Low*) were a gift from Dr Denise Connolly (Fox Chase Cancer Center, Philadelphia, PA, USA), and housed in the COSR at RPCCC. These non-tumor-prone mice express the TAG protein in epithelial cells lining the fallopian tubes under transcriptional control of the Müllerian inhibiting substance type II receptor gene promoter.^13^ All animal studies were performed in compliance with the guidelines established by the Institutional Animal Care and Use Committee under approved protocols. The TAG-expressing MOVCAR 5009 ovarian carcinoma cell line, transduced with a retroviral construct encoding the firefly luciferase gene (pWZL-Luc) for in vivo imaging,^13^ was kindly provided by Dr Denise Connolly (Fox Chase Cancer Center). MOVCAR 5009 cells were cultured in DMEM (10-013-CV, Corning, NY, USA) supplemented with 10% fetal bovine serum (FBS; 35-010-CV, Corning), 5 μg/mL gentamicin sulfate (30-005-CR, Corning), and maintained at 37 °C with 5% CO_2_. The MOVCAR 5009 cell line was authenticated at the American Type Culture Collection (ATCC; Manassas, VA, USA) using short tandem repeat profiling.

### Oncolytic vaccinia viruses

The OVs used in the study were of the Western Reserve strain with disrupted thymidine kinase (*TK*) and vaccinia growth factor (*VGF*) genes for enhanced cancer cell specificity. The generation and characterization of the virus expressing the Fc portion of murine IgG2a (OV-Fc) and CXCR4 antagonist in the context of the Fc portion of murine IgG2a (OV-CXCR4-A) have been described previously.^12^

### In vivo studies

SCID mice (*n* = 5/group) and *TgMISIIR-TAg-Low* mice (*n* = 5/group) were injected intraperitoneally (i.p.) with 5 × 10^6^ MOVCAR 5009 cells and treated i.p. with 5 × 10^7^ PFU of OV-Fc or OV-CXCR4-A 10 days after the tumor challenge, while untreated mice served as controls. For the ACT, spleens from naïve B6.Cg-Tg(TcraY1, TcrbY1)416Tev/J mice were collected, homogenized, and filtered through a 70-µm cell strainer (Fisher Scientific, Waltham, MA, USA) into ammonium-chloride-potassium lysis buffer (118-156-101, Quality Biological, Gaithersburg, MD, USA) to lyse erythrocytes. Splenocytes were washed twice in cold RPMI 1640 media (10-040-CV, Corning) and used for isolation of TCR_TAG_ T cells using the Pan T Cell Isolation Kit II (130-095-130, Miltenyi Biotech, Gaithersburg, MD, USA). Five × 10^6^ TCR_TAG_ T cells, were injected intravenously (i.v.) into MOVCAR 5009-bearing SCID and *TgMISIIR-TAg-Low* mice 13 days after tumor challenge or 3 days after oncolytic virotherapy treatment. Tumor growth was monitored by bioluminescence imaging, and signals were determined by IVIS Spectrum In Vivo Imaging System (PerkinElmer, Waltham, MA, USA) after i.p. injection of 200 µL D-Luciferin (150 mg/kg body weight; LUCK-1G, Gold Biotechnology, St Louis, MO, USA) following the manufacturer’s protocol. The values for average radiance (photons/sec/cm^2^/sr) in regions of interest (ROIs) were determined in the Living Image v.4.7.3 Software for IVIS Spectrum.

### Immunohistochemistry

Immunohistochemical staining was done on the omental tumor sections harvested from MOVCAR 5009-bearing SCID mice 10 days after OV treatments. In brief, samples were placed in 10% neutral buffered formalin for 24 h, dehydrated, and embedded in paraffin. Formalin-fixed, paraffin-embedded (FFPE) sections (4 µm thick) were stained using a rat anti-mouse antibody specific for CD31 (clone: SZ31, DAI-310; Dianova, Eching, Germany) at 1:35 dilution for 40 min, followed by incubation with rabbit anti-rat IgG (ab102248; Abcam, Cambridge, UK) for 30 min, and Rabbit EnVision^+^ (K4003; Agilent Technologies, Santa Clara, CA, USA) for 30 min. Diaminobenzidine (DS9800; Leica Biosystems, Wetzlar, Germany) was applied for 10 min, and slides were counterstained with hematoxylin for 8 min. Slides were scanned by the Aperio AT2 Slide scanning system, and data were analyzed with the Aperio ImageScope 12.3.3 Software (Leica Biosystems). MVD was evaluated by enumerating the number of CD31^+^ endothelial clusters within an ROI, and microvessel diameter was measured at the vessel’s largest width as described.^4^^,^^12^

### The multispectral immunofluorescence imaging

#### Sample preparation and staining

The multispectral immunofluorescence (mIF) staining on FFPE omental tumor sections was performed by the Advanced Tissue Imaging Shared Resource at RPCCC using the Opal 6-Plex Detection Kit (NEL821001KT, AKOYA Biosciences, Marlborough, MA, USA) as described.^15^ In brief, FFPE 4-µm sections were cut and placed on charged slides. Slides were dried at 65 °C for 2 h. After drying, the slides were placed on the BOND RXm Research Stainer (Leica Biosystems) and deparaffinized with BOND Dewax solution (AR9222, Leica Biosystems). The mIF staining process involved serial repetitions of the following for each biomarker: epitope retrieval/stripping with ER1 (citrate buffer pH 6, AR996, Leica Biosystems) or ER2 (Tris-EDTA buffer pH 9, AR9640, Leica Biosystems), blocking buffer (AKOYA Biosciences), primary antibody, Opal Polymer HRP secondary antibody (AKOYA Biosciences), and Opal Fluorophore (AKOYA Biosciences). Spectral DAPI (AKOYA Biosciences) was applied once slides were removed from the BOND. They were cover-slipped using an aqueous method and Diamond antifade mounting medium (Invitrogen, Thermo Fisher Scientific, Waltham, MA, USA). The mIF panels consisted of the antibodies as detailed in Table S1.

#### Tissue imaging and analysis

Slides were imaged using the PhenoImager HT (AKOYA Biosciences). Further analysis of the slides was performed with inForm Software v.2.6.0 (AKOYA Biosciences). Whole slides were first scanned in an unmixed view, and representative ROIs were selected for acquisition under the guidance of a pathologist. These ROIs were then rescanned to achieve full spectral unmixing. A representative subset of the unmixed ROIs was used to train tissue and cell segmentation. An algorithm was subsequently created using a machine-learning technique, where the operator selects positive and negative cell examples for each marker. These algorithms were then batch-applied to additional ROIs for further analysis. The RStudio plugin phenoptrReports was used to extract phenotype counts from the resulting data tables.

### Spatial transcriptomics

#### Sample preparation

Spatial transcriptomics (ST) analysis was completed on FFPE sections prepared from omental tumors of MOVCAR 5009-bearing SCID mice 10 days after OV treatments using the 10x Genomics Visium platform by the Genomics Shared Resource at RPCCC. FFPE blocks were stained with H&E, imaged for pathological review, sectioned at 5 µm, and trimmed to fit in the capture area (6.5 mm × 6.5 mm) of the Visium Spatial slides (10x Genomics, Pleasanton, CA, USA). Each area contains an array of ∼5000 spots (55 µm in diameter). After tissue mounting on Visium slides, H&E images were captured for downstream data analysis. The tissue areas of interest were identified by a pathologist based on histologically well-organized CD31-expressing tumor vasculature. The 10x Visium library preparation was performed using Visium Spatial Transcriptomic v.1 Kit following the manufacturer’s recommendations. In brief, the RNAs within the tissue were hybridized to the mouse whole transcriptome probe panel, and the hybridized probes were captured on the Visium slides. Captured probe products were then extended using the unique molecular identifier (UMI), Spatial Barcode, and partial Read 1 adapter to generate spatially barcoded and ligated probe products. The ligated products were released from the slide, and qPCR was used to determine the sample index PCR cycles. Final libraries were generated by PCR using sample indexing primers. The resulting libraries were evaluated using D1000 Screen Tape on the TapeStation 4200 (Agilent Technologies) and quantified using the KAPA Biosystems qPCR quantitation kit for Illumina. They were then pooled, denatured, and diluted to 300 pM (picomolar) with 1% PhiX control library added. The resulting pool was then loaded into the appropriate NovaSeq Reagent cartridge, followed by sequencing on a NovaSeq6000 according to the manufacturer’s protocol (Illumina, San Diego, CA, USA). Once sequencing was completed, tissue images taken after H&E staining on the Visium slide were used to align the gene expression from the spatial barcodes unique to each location in the capture area during data analysis.

#### 10x genomics Visium data analysis

For the 10x Genomics Visium analysis, mapping results (binary alignment and map [BAM] files) and quantification matrices were generated using Space Ranger v.1.3.1 software with the mouse mm10 genome and GENCODE annotation database. Filtered gene-barcode matrices containing barcodes with UMI counts that passed the cell detection algorithm were used for downstream analysis. Downstream analyses were performed using the Seurat single-cell data analysis R package. Five to 7 ROIs on slides prepared with FFPE tissue, containing well-organized CD31-expressing tumor vasculature networks, were identified by a pathologist within each omental tumor section. Spots within individual ROIs were selected based on the expression of *Pecam1* and *Cd3d* transcripts. A total of 84 spots were analyzed for untreated tumors, 50 spots for ACT-treated tumors, 134 spots for OV-Fc/ACT–treated tumors, and 87 spots for OV-CXCR4/ACT–treated tumors. Normalized and scaled UMI counts were calculated using the SCTransform method. Differentially expressed genes between clusters and samples were identified using the FindMarkers function with the Wilcoxon rank-sum test from Seurat, with Benjamini–Hochberg adjusted *P* values calculated for multiple test correction. The average log_2_ fold change ordered gene lists generated from FindAllMarkers function were used for gene set enrichment analysis (GSEA), which was carried out using fgsea R package. Activation/effector and dysfunction scores were calculated using the AddModuleScore method from Seurat with selected genes in each functional category. Violin plots were generated using the geom_violin function from the ggplot2 R package, and *P* values were added using the stat_compare_means function from the ggpubr R package with the Wilcoxon rank-sum test. The normalized gene expression data matrix was used to generate heatmaps using the pheatmap R package, with the data row-scaled and centered. The frequency of each immune cell type was calculated as the number of spots expressing the corresponding cell type marker divided by the total number of spots per treatment group. Volcano plots for differential gene expression were generated using the EnhancedVolcano R package. Venn diagrams were generated with the ggvenn R package using differentially expressed gene analysis results.

### Single-cell RNA sequencing

#### Sample preparation

The single-cell RNA sequencing (scRNA-seq) analysis was performed on single-cell suspensions prepared from tumor-draining lymph nodes (tdLNs) of MOVCAR 5009-bearing SCID mice (*n* = 4 mice/group) 10 days after oncolytic virotherapy treatments. The lymph nodes were harvested and homogenized, and obtained cells were resuspended in 0.04% bovine serum albumin (BSA; A9418, Sigma-Aldrich, Burlington, MA, USA) in PBS without calcium and magnesium (PBS; 21-031-CV, Corning), passed through a 70-μm cell strainer (Fisher Scientific) to receive single-cell suspension and washed 3 times in prechilled 1% BSA in PBS. Single-cell gene expression libraries were created using the 10x Genomics Chromium Next GEM Single Cell 3′ Kit v.3.1 (10x Genomics). To evaluate the viability and number of cells, as well as the absence of clumps and debris in single-cell suspensions, trypan blue and a Countess FL automated cell counter (Thermo Fisher Scientific) were used. Subsequently, samples from different experimental groups (ACT, OV-Fc/ACT, OV-CXCR4-A/ACT) were loaded into the Chromium Controller (10x Genomics), targeting the capture of approximately 5,000 cells for each sample, followed by reverse transcription and cDNA amplification. This full-length amplified cDNA was then used to generate transcriptome libraries by enzymatic fragmentation, end-repair, A-tailing, adapter ligation, and PCR to add Illumina-compatible sequencing adapters. Evaluation of the obtained libraries was achieved on D1000 screen tape using a TapeStation 4200 (Agilent Technologies) and quantification using the Kapa Biosystems qPCR Quantitation Kit for Illumina. The libraries were denatured, diluted to 300 pM with 1% PhiX control library, loaded into the NovaSeq reagent cartridge, and sequenced on a NovaSeq6000 according to the manufacturer’s protocol (Illumina). The received sequencing data from the 10x Genomics libraries were processed in Cellranger v.7.0.0 Software (10x Genomics).

#### scRNA-seq analysis

For the scRNA-seq analysis, mapping results (BAM files) and quantification matrices were generated using Cell Ranger v.7.0.0 Software with the mouse mm10 genome and GENCODE annotation database. Filtered gene-barcode matrices, which contain barcodes with UMI counts that passed the cell detection algorithm, were used for further analysis. Downstream analyses were performed using the Seurat single-cell data analysis R package. Cells with very low or high RNA feature content (<500 or >7,500 genes detected) or higher mitochondrial RNA content (>15%) were filtered out to exclude empty cells and doublets. Normalized and scaled UMI counts were calculated using the SCTransform method. Subsequently, dimension reductions, including principal component analysis, uniform manifold approximation and projection (UMAP), and t-distributed stochastic neighbor embedding, were carried out using highly variable genes. Cell clusters were identified using shared nearest neighbor–based clustering on the first 12 principal components. Cell cluster annotation was performed using the SingleR packages and the ImmGen reference database from the celldex R package. Differentially expressed genes between clusters and samples were identified using the FindMarkers function with the Wilcoxon rank-sum test in Seurat. Normalized gene expression data matrix was used to generate heatmaps using the pheatmap R package, with data row-scaled and centered.

### Flow cytometry

Flow cytometry analyses were performed on single-cell suspensions obtained from several sources: splenocytes after T-cell separation from naïve B6.Cg-Tg(TcraY1, TcrbY1)416Tev/J mice; omental tumors; inguinal tdLNs of MOVCAR 5009-bearing SCID mice 10 days after oncolytic virotherapy; and omental tumors of MOVCAR 5009-bearing *TgMISIIR-TAg-Low* mice 10 days after OV treatment. Tumor samples were processed as described previously.^16^ In brief, omenta were harvested, minced, and digested with 0.4% collagenase (C0130, Sigma-Aldrich) for 30 min at 37 °C. The resulting cell suspensions were passed through a 70-μm cell strainer to eliminate large aggregates, centrifuged at 250 × *g* for 5 min at 4 °C, and washed in buffer (2% FBS and 0.1% NaN_3_ in PBS). For staining, single-cell suspensions were incubated with an anti-mouse CD16/CD32 Fc blocking antibody (553142, BD Biosciences, Franklin Lakes, NJ, USA) for 20 min at 4 °C, followed by extracellular staining with anti-mouse fluorochrome-conjugated antibodies for 30 min at 4 °C in the dark. All antibodies were purchased from BD Biosciences or BioLegend (San Diego, CA, USA), as detailed in Table S2. For the exclusion of dead cells, Live/Dead Fixable Aqua Stain was used according to the manufacturer’s instructions (L34966, Thermo Fisher Scientific). Samples were acquired with the LSR Fortessa flow cytometer (BD Biosciences) and FACSDiva Acquisition Software (BD Biosciences). Data analysis was performed using WinList 3D 9.0.1 (Verity Software House, Topsham, ME, USA). During analysis, doublets were excluded using forward scatter height (FSC-H) versus forward scatter area (FSC-A).

### Statistical analysis

Statistical analyses were performed using GraphPad Prism 10 (GraphPad Software, San Diego, CA, USA) and R software. Two-way ANOVA (analysis of variance) with Tukey multiple comparisons test was used to determine significant differences in tumor growth between groups. Kaplan–Meier survival plots were generated for tumor-challenged groups of mice, and statistical differences in survival periods between groups were calculated using the log-rank Mantel–Cox method. The Wilcoxon rank-sum test was used to test the differences between samples for mIF, Visium spatial transcriptomic, and scRNA-seq data, and flow cytometry analyses. Fisher exact test was used to compare cell composition between samples. The Pearson correlation between activation/effector scores and dysfunction scores was tested using the stat_cor method from the ggpubr package. Data are presented as noted in the figure legends. For box plots, the line inside the box represents the median, while the lower and upper hinges correspond to the first and third quartiles (the 25th and 75th percentiles). The upper whisker extends from the hinge to the largest value within 1.5 × IQR (interquartile range) of the hinge, and the lower whisker extends to the smallest value within 1.5 × IQR. Data points beyond the whiskers are considered “outliers” and are plotted individually. The threshold for statistical significance was set at *P* < 0.05.

## Results

### OV-CXCR4-A treatment increases the efficacy of adoptively transferred TCR_TAG_ T cells in MOVCAR 5009-challenged SCID mice

**Figure 1.**
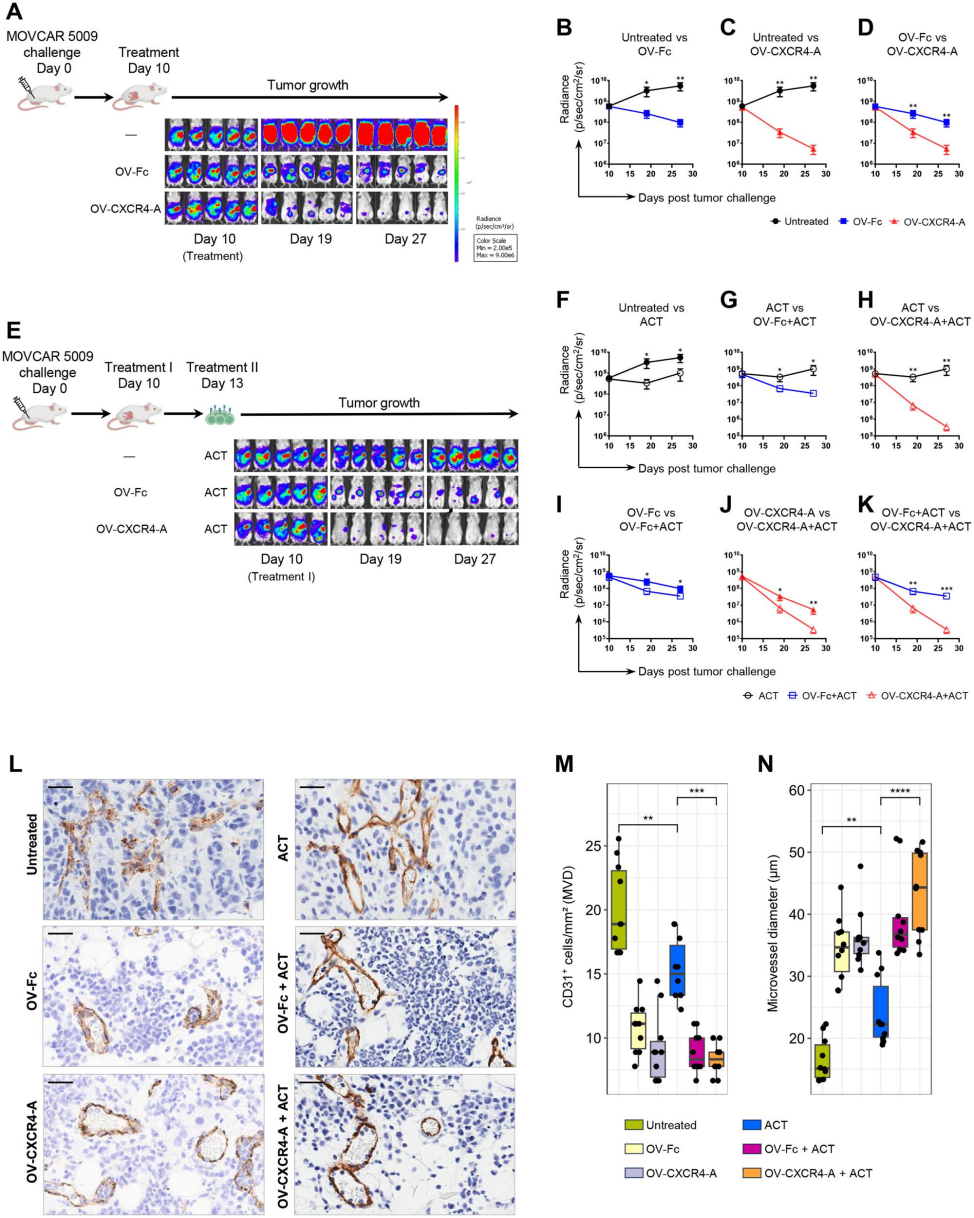
The effect of OV and ACT treatments on the progression of MOVCAR 5009 tumor growth and changes in the tumor vasculature. (A) Experimental scheme of the OV treatment (upper panel). Female SCID mice (*n* = 5/group) were injected i.p. with 5 × 10^6^ TAG^+^ MOVCAR 5009 cells and treated i.p. with 5 × 10^7^ PFU of OV-Fc or OV-CXCR4-A 10 days later. Control mice were treated with PBS. Tumor progression was monitored by bioluminescence imaging on days 10, 19, and 27 post–tumor challenge (lower panel). (B–D) Tumor growth curves in mice after different treatments. Individual data points are represented as mean ± SD of 3 independent experiments. Statistical analysis was performed using 2-way ANOVA. *P *<* 0.05; **P *<* 0.01. (E) Experimental scheme of ACT monotherapy and combination treatments. For ACT, TCR_TAG_ T cells purified from splenocytes of transgenic B6.Cg-Tg(TcraY1, TcrbY1)416Tev/J mice using the Pan T-cell isolation kit II were injected i.v. into tumor-bearing mice (5 × 10^6^ cells/mouse) 13 days after tumor challenge or 3 days after OV treatment (upper panel). Tumor progression was monitored by bioluminescence imaging on days 10, 19, and 27 post–tumor challenge (lower panel). (F–K) Tumor volume curves in mice after different treatments. Individual data points are represented as mean ± SD of 3 independent experiments. Statistical analysis was performed using 2-way ANOVA. *P *<* 0.05; **P *<* 0.01; ***P *<* 0.001. (L) IHC staining of omental tumor sections with CD31-specific mAb from one representative ROI. Scale bars: 30 µm. (M and N) Box plots displaying tumor MVD (M) and microvessel diameter (N) across independent ROIs (10/group). The results are from 2 independent experiments. *P* values were calculated using a 2-sided Wilcoxon rank-sum test. **P *<* 0.01; ***P *<* 0.001; ****P *<* 0.0001.

To address the antitumor effect of oncolytic virotherapy when delivered alone or in combination with ACT of tumor-specific TCR_TAG_ T cells, we used a metastatic TAG^+^ MOVCAR 5009 ovarian tumor grown orthotopically in SCID mice. This model overcomes the limitations of a small fraction of tumor antigen (Ag)–specific T cells with diverse TCR repertoires in immunocompetent mice, allowing us to focus on the Ag-specific T cell–tumor interaction in the absence of irrelevant endogenous bystanders.^17^ As depicted in Figure 1A (upper panel), MOVCAR 5009 cells (5 × 10^6^/mouse) were injected i.p. into mice, which were then either left untreated or treated 10 days later with OV-Fc or OV-CXCR4-A delivered i.p. at 5 × 10^7^ plaque-forming units (PFU)/mouse. Inhibition of tumor growth quantified by bioluminescence imaging (Fig. 1A, lower panel) revealed that although both viruses significantly inhibited tumor growth and increased survival compared to untreated mice (Fig. 1B, C; Fig. S1A, B), OV-CXCR4-A treatment exhibited higher efficacy compared to its control counterpart (Fig. 1D; Fig. S1C). This could be related to the direct antitumor activity of CXCR4-A through induction of apoptosis, antibody-dependent cellular cytotoxicity, and complement-dependent cytotoxicity.^10^^,^^12^^,^^18^ For the adoptive transfer, TCR_TAG_ T cells purified from splenocytes of transgenic B6.Cg-Tg(TcraY1, TcrbY1)416Tev/J mice using the Pan T-cell isolation kit II were injected i.v. into tumor-bearing mice (5 × 10^6^ cells/mouse) 13 days after tumor challenge or 3 days after OV treatment (Fig. 1E, upper panel). The timing of the adoptive transfer was chosen based on the kinetics of the transferred cells, which showed peak efficacy around day 3 following viral delivery (data not shown). This could be linked to the transient decrease in vessel perfusion that occurs within the first 48 hours of OV delivery and subsequent revascularization beginning around day 10.^19^ The purified immune cells transferred consisted of 77% to 83% CD8^+^ T cells and 13% to 23% CD4^+^ T cells (Fig. S2). Among other CD45^+^ cells, 5% to 8% comprised CD19^+^ B cells, 0.9% to 3% myeloid cells, and 0.1% to 0.3% NK.1.1^+^ natural killer (NK) cells. CD45^−^ cells represented less than 3% of the total population. Tumor-bearing mice treated with ACT 13 days after tumor inoculation served as controls. Kinetics analysis of tumor growth, quantified by bioluminescence imaging (Fig. 1E, lower panel), revealed a short-lasting inhibition following ACT monotherapy (Fig. 1F) and increased survival (Fig. S1D), indicating antitumor activities of TCR_TAG_ T cells, albeit with moderate efficacy. The ACT/OV treatment combinations increased both the antitumor efficacy of ACT (Fig. 1G, H; Fig. S1E, F) as well as monotherapies with respective viruses (Fig. 1I, J; Fig. S1G, H) with a more profound antitumor effect mediated by OV-CXCR4-A than the OV-Fc combination (ie ∼100- vs ∼10-fold reduction of the tumor burden on day 19). The slower kinetics of tumor growth after OV-CXCR4-A/ACT compared to OV-Fc/ACT treatment (Fig. 1K) was also reflected by longer survival periods in the former group of animals (Fig. S1I).

Because MOVCAR 5009 tumor cells grow orthotopically in non-tumor-prone C57BL/6 *TgMISIIR-TAg-Low* transgenic mice, which express the TAG protein in epithelial cells lining the fallopian tubes under transcriptional control of the Müllerian inhibiting substance type II receptor gene promoter,^11^^,^^14^ we performed additional experiments using MOVCAR 5009-bearing *TgMISIIR-TAg-Low* transgenic mice. As shown in Figure S3A and B, the OV-CXCR4-A/ACT treatment resulted in the most pronounced inhibition of tumor growth and the longest survival compared to animals treated with ACT alone or OV-Fc/ACT combination. Given that identical cancer cells and tumor-specific T cells were injected into all animals, these results indicated that the TME impacted the T-cell effector differentiation through immune modulation.

### Restoration of aberrant tumor blood vessels after OV/ACT combinations

**Figure 2.**
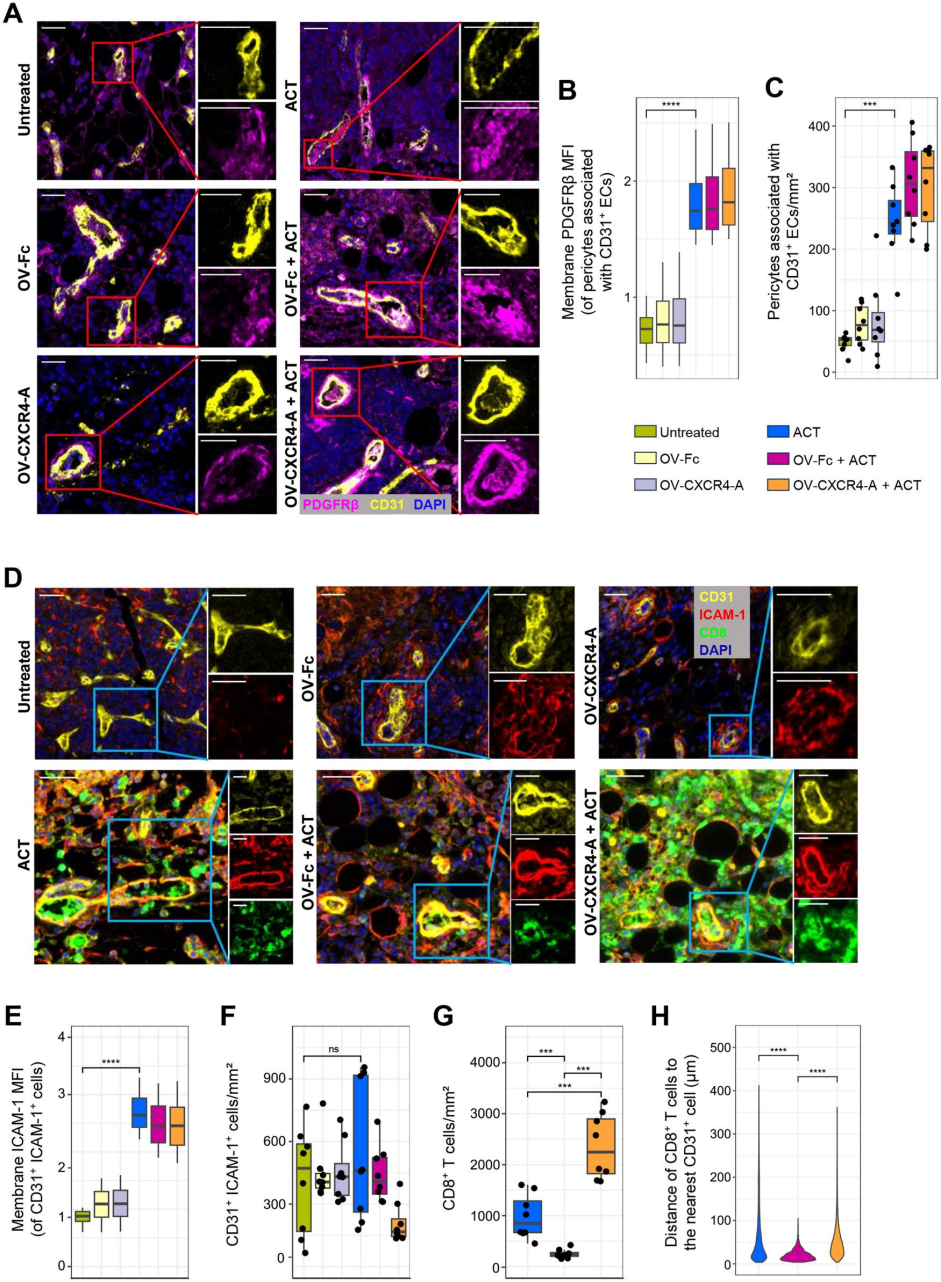
Changes in the tumor vasculature following OV and ACT treatments. (A) mIF images from representative ROIs of MOVCAR 5009 omental tumors showing treatment-mediated changes in the expression of CD31 on tumor vascular ECs (left panel) and PDGFRβ on pericytes associated with CD31^+^ ECs (right panel). The nuclei were visualized by DAPI staining. CD31-Opal 570; PDGFRβ-Opal 520. Scale bars: 30 µm. (B) Box plot of PDGFRβ expression levels on pericytes associated with vascular ECs calculated from independent ROIs (8/group). The results represent 2 independent experiments. MFI, mean fluorescence intensity. (C) Density of EC-associated pericytes calculated from the same ROIs. (D) Multispectral IF images form representative ROIs showing changes in CD31 and ICAM-1 expression on the tumor vascular ECs (upper panel), and CD8 expression on adoptively transferred CD8^+^ TCR_TAG_ T cells (lower panel). CD31-Opal 570; ICAM-1-Opal 620; CD8-Opal 690. Scale bars: 30 µm. (E) Box plot of ICAM-1 expression on CD31^+^ ECs across independent ROIs (8/group). (F) Box plot displaying the MVD of CD31^+^ICAM-1^+^ ECs from the same ROIs. (G) Accumulation of CD8^+^ T cells in the perivascular area of omental tumor metastases. (H) Colocalization of CD8^+^ T cells relative to the nearest CD31^+^ vascular ECs. The results are from 8 independent ROIs per group, performed in duplicate. *P* values were calculated using a 2-sided Wilcoxon rank-sum exact test. ***P *<* 0.001; ****P *<* 0.0001; ns, nonsignificant.

As the abnormal tumor vasculature affects the infiltration of immune cells and promotes immune suppression through the synthesis of proangiogenic receptors and factors,^20^ we examined changes in microvessel density (MVD) and vessel size in the omental tumors 10 days after treatment initiation. The immunohistochemical staining of tumor sections from untreated mice with anti-PECAM-1/CD31 mAb revealed a disorganized blood vessel network with high MVD and small luminal diameters compared to the OV-treatment groups (Fig. 1L, left panel). Virotherapy treatment with either OV-Fc or OV-CXCR4-A reduced the MVD by approximately 2-fold and increased luminal diameters (Fig. 1M, N), supporting the reported pruning ability of OVs by infecting and eliminating proliferating tumor vascular ECs.^6^ These findings align with our previous studies in 4T1 breast carcinoma-bearing BALB/c mice, which showed reduced MVD in tumor sections, particularly in the core regions, following OV treatment compared with controls^12^ (Fig. 2). We also observed decreases in MVD after ACT (Fig. 1L [right panel], M, and N), though the effect was less prominent than that mediated by OV treatment. The latter observation aligns with the angiostatic effect of IFN-γ, which directly inhibits the proliferation and migration of ECs.^21^

We next examined the fortification of the blood vessels by pericyte coverage of ECs,^22^ the lack of which constitutes one of the inhibitory mechanisms limiting T-cell transendothelial migration.^23^ Paraffin sections of omental tumors from all treatment groups were analyzed by mIF imaging for CD31^+^ vascular ECs and cells associated with ECs that express platelet-derived growth factor receptor β (PDGFRβ) as a marker of pericytes.^24^ As shown in Figure 2A (left panel), the blood vessels in untreated tumors or tumors treated with either OV-Fc or OV-CXCR4-A were poorly covered by EC-associated PDGFRβ^+^ pericytes (Fig. 2B, C), indicating that OV treatments alone did not contribute to the vascular normalization process. In contrast, PDGFRβ was highly expressed on pericytes after ACT treatment, either alone or in combination with OV (Fig. 2A [right panel], B, and C), and the improved restoration of aberrant tumor vasculature with ACT alone or in combination with OV treatment was accompanied by increased expression of intercellular adhesion molecule ICAM-1 (CD54).^25^ The ICAM-1 expression levels in untreated or virotherapy-treated tumors (Fig. 2D, upper panel) were lower compared to those receiving ACT alone or in combination with OV (Fig. 2D [lower panel], E, and F), indicating that the adoptively transferred T cells alone contributed to tumor vasculature normalization. Interestingly, however, despite similar ICAM-1 expression in mice receiving ACT treatment alone or in combination, the trafficking of CD8^+^ TCR_TAG_ T cells into tumors differed (Fig. 2D, lower panel). As shown in Figure 2G, the lowest penetration of CD8^+^ TCR_TAG_ T cells into the perivascular tumor areas was observed after OV-Fc treatment (248.7 ± 31.5 cells/mm^2^) compared to approximately 4- and 10-fold higher numbers in the untreated and OV-CXCR4-A–treated tumors (978.7 ± 153.6 cells/mm^2^ and 2,355.6 ± 225.8 cells/mm^2^, respectively). T cells were predominantly localized within the 50-µm area surrounding the blood vessels after OV-Fc treatment, whereas in untreated and OV-CXCR4-A–treated tumors, they migrated throughout the perivascular areas (Fig. 2H).

### Transcriptomic profiles of endothelial adhesion molecules, angiogenic receptors, and growth factors after ACT and OV treatments

**Figure 3.**
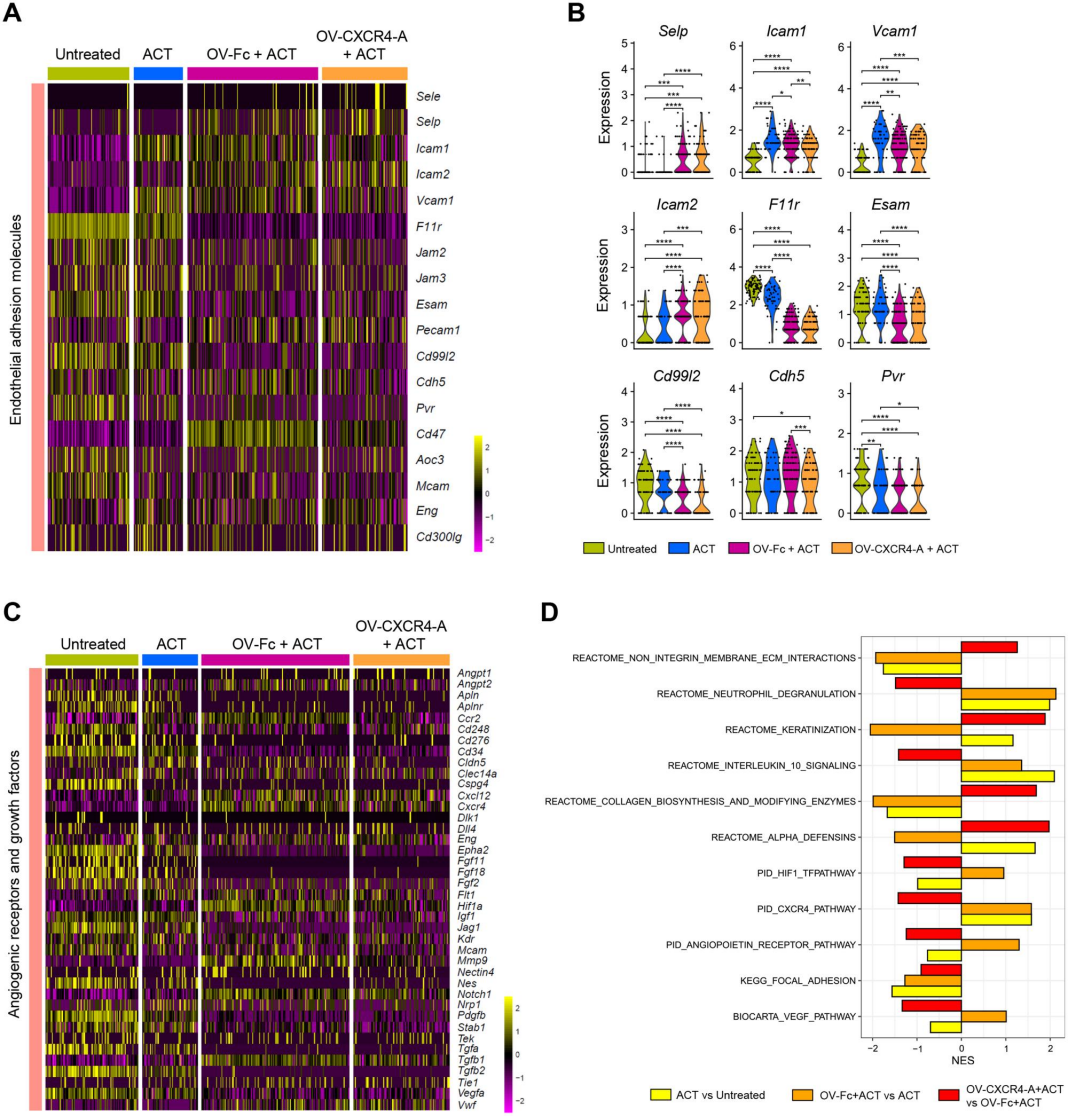
Treatment-induced differences in transcriptomic profiles of endothelial adhesion molecules (EAMs), angiogenic receptors, and growth factors. (A) Heatmap displaying the expression of genes encoding EAMs on the tumor vascular endothelium. The number of spots analyzed was 84 for untreated tumors, 50 for ACT-treated tumors, 134 for OV-Fc and ACT-treated tumors, and 87 for OV-CXCR4 and ACT-treated tumors. (B) Violin plots comparing the expression of indicated genes within the EAM population among the treatment groups. *P* values were calculated using a 2-sided Wilcoxon rank-sum exact test. *P *<* 0.05; **P *<* 0.01; ***P *<* 0.001; ****P *<* 0.0001. (C) Heatmap presenting the expression of the genes encoding angiogenic receptors and growth factors in the perivascular TME of omental metastases. (D) Bar plot of GSEA of ACT vs untreated, OV-Fc + ACT vs ACT, and OV-CXCR4-A + ACT vs OV-Fc + ACT, showing NES. NES, normalized enrichment score.

To better understand the mechanisms responsible for the differences in the observed accumulation of the adoptively transferred T cells, we performed ST analyses of the perivascular TME using omental tumor sections collected 10 days after treatment initiation. ROIs within FFPE tumor tissues were selected based on the presence of well-organized CD31^+^ tumor vasculature networks (Fig. S4A). Although this selection strategy may introduce bias by excluding nonvascularized areas containing CD31-expressing cells that are not associated with vessel lining,^25^^,^^26^ our rationale was based on ensuring detectable *Pecam1* and *Cd3d* transcript expression within the 55-μm spots of each ROI, which was required for transcriptomic characterization of T-cell trafficking into the perivascular TME. Analysis of EC adhesion receptors expression involved in leukocyte extravasation (Fig. 3A, B) revealed that transcription levels of endothelial E/P selectin genes (*Sele* and *Selp*)^25^ were low across analyzed tumor sections, with *Selp* increasing only after combination treatments. Consistent with the mIF imaging, *Icam1* expression was upregulated in all treatment groups, along with vascular cell adhesion molecule 1 (*Vcam1*), reaching the highest levels after ACT. Surprisingly, the transcriptomic profiles of EC adhesion molecules involved in transendothelial migration/diapedesis processes,^25^ including junctional adhesion molecules (*Jam1/F11r*), *Cd99l2*, and *Pvr*, were higher in untreated and TCR_TAG_ T cell–treated tumors compared to those receiving the combination treatments. These results might reflect treatment-mediated changes in the dominant cell types surrounding endothelial cell junctions, which involve different modifications of VE-cadherin (*Cdh5*) depending on the type of stimulus and the purpose of junction opening.

The expression of several genes promoting angiogenesis and angiogenic growth factors was reduced following the adoptive transfer of TCR_TAG_ T cells and was further downregulated in mice treated with OV-Fc/ACT and OV-CXCR4-A/ACT combinations. Notably, the OV-CXCR4-A/ACT treatment exhibited superior angiostatic activity compared to OV-Fc/ACT, as evidenced by the marked downregulation of *Angpt2*, *Ccr2*, *Hif1a*, *Mmp9*, *Stab1*, *Tgfb1*, and *Cxcr4* (Fig. 3C; Fig. S4B). These findings likely reflect the inhibitory activity of the soluble CXCR4-A protein released from virally infected tumor cells.^12^ Transcriptomic differences in angiogenesis-associated genes across treatment groups were further validated by GSEA. As shown in Figure 3D, ACT alone or in combination with OV-CXCR4-A led to downregulation of HIF1 transcription factor, CXCR4, angiopoietin receptor, and VEGF signaling pathways, along with upregulation of integrin-ECM pathways, which are critical for angiogenesis. Interestingly, OV-Fc/ACT produced effects opposite to those of OV-CXCR4-A/ACT, suggesting that these treatment combinations act through distinct, largely nonoverlapping signaling pathways or target different components within the same pathway. This pattern extended to other pathways, including neutrophil degranulation, keratinization, IL-10 signaling, collagen biosynthesis, and α-defensin pathways, highlighting the unique immunomodulatory effects of OV-CXCR4-A/ACT on perivascular niche reprogramming.

### The impact of ACT and OV treatment on cellular composition of the perivascular TME

Analyses of the perivascular TME using ST and mIF imaging revealed that cancer cells were the predominant population in omental metastases of both untreated and ACT-treated mice, as indicated by elevated expression of keratin 8 (*Krt8*) and accumulation of KRT8^+^ cells (Fig. S5A and S5B, respectively). In contrast, combination therapies resulted in a marked reduction in KRT8^+^ cells. The mIF imaging further showed that in untreated mice, clusters of KRT8^+^ tumor cells were closely associated with tumor vasculature. These clusters became progressively less dense following ACT monotherapy and were reduced to small aggregates after combination treatments (Fig. S5C). Differences were also observed in the colocalization of tumor vasculature and KRT8^+^ cells relative to collagen 1α1 (COL1α1) protein fibers, known for adverse effects on T-cell trafficking.^27^ In untreated tumors, tightly organized and parallel fibers of COL1α1 were found around blood vessels or at the tumor–stroma interface, which contrasted with loose networks observed in the treatment groups (Fig. S5C).

**Figure 4.**
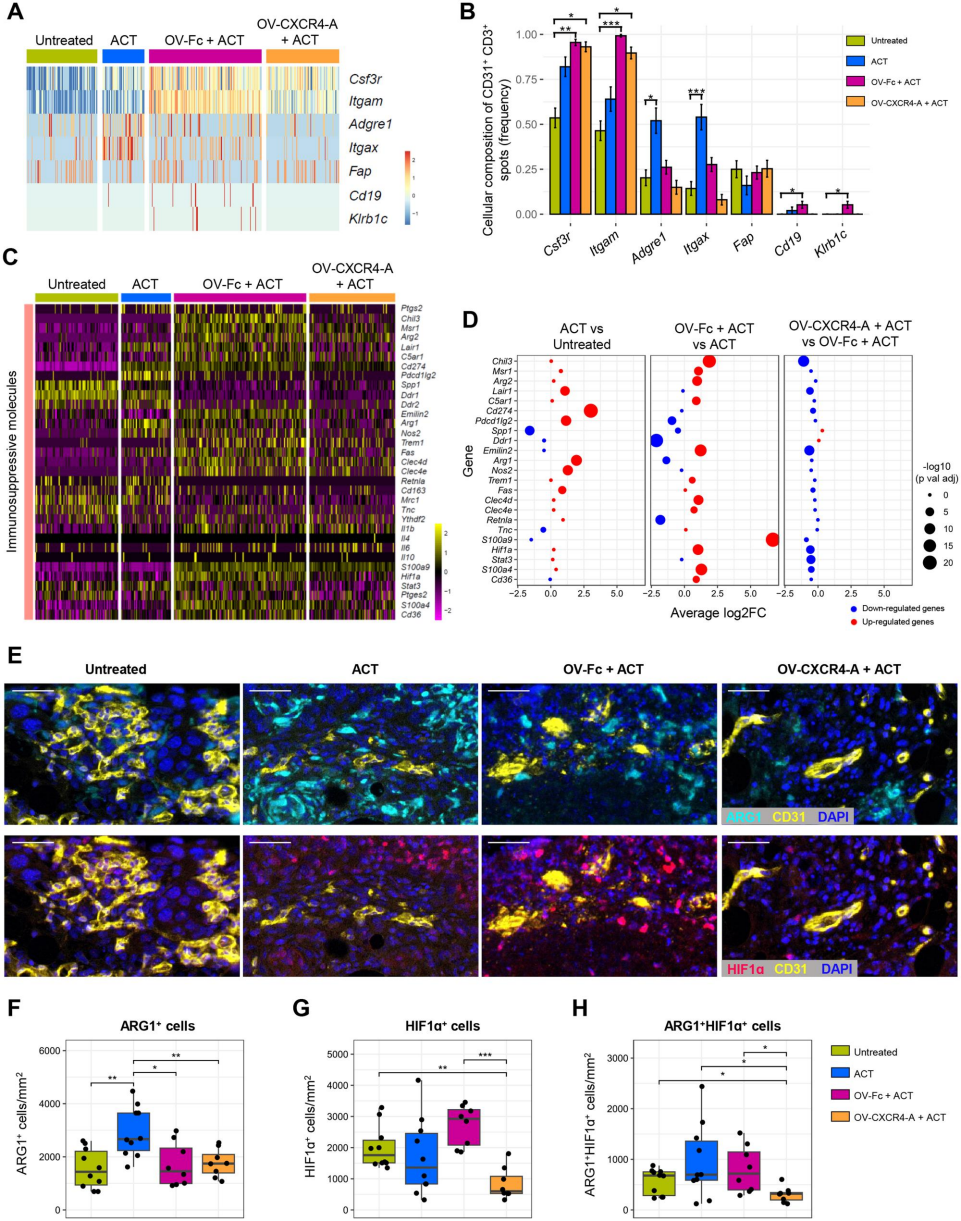
Differences in the influx of myeloid cells and expression of immunosuppressive molecules in the perivascular TME after ACT and OV/ACT combination treatments. (A) Heatmap displaying the row-scaled expression of selected genes linked to various cell populations infiltrating the perivascular TME in omental tumor sections analyzed via spatial transcriptomics. Markers include CSF3R (*Csf3r*) and CD11b (*Itgam*) for neutrophils, F4/80 (*Adgre1*) for TAMs, CD11c (*Itgax*) for monocytes/DCs, fibroblast activation protein (*Fap*) for CAFs, CD19 (*Cd19*) for B cells, and NK1.1 (*Klrb1c*) for NK cells. (B) Frequency of cells infiltrating the perivascular niches. The frequency of each cell type was calculated as the number of spots expressing the corresponding cell type marker divided by the total number of spots per treatment group. Standard deviation was calculated assuming a binomial distribution for each cell type. Data are presented as mean ± SD and analyzed using Fisher exact test. * *P* *<* 0.05; ** *P* *<* 0.01; ***P *<* 0.001. (C) Heatmap showing normalized gene expression associated with immunosuppressive myeloid cell subsets. (D) Dot plot illustrating differences in immunosuppressive gene expression among treatment groups using Visium transcriptome data. Enhanced expression (red) and reduced expression (blue) dots represent individual genes with the dot size reflecting the negative log_10_-transformed Benjamini–Hochberg-adjusted *P* values. *n* = 84 spots for untreated tumors, *n* = 50 spots for ACT-treated tumors, *n* = 134 spots for OV-Fc/ACT–treated tumors, *n* = 87 spots for OV-CXCR4/ACT–treated tumors. (E) Multispectral IF images from representative ROIs showing treatment-mediated changes in the colocalization of CD31^+^ vascular ECs and ARG1^+^ cells (upper panel) and HIF1α^+^ cells (lower panel). Omental tumor sections were stained with anti-CD31, -ARG1, and -HIF1α mAbs, counterstained with DAPI to visualize nuclei, and analyzed using the PhenoImager HT. CD31-Opal 480; ARG1-Opal 690; HIF1α-Opal 520. The same ROIs as in Figure S4C are presented. Scale bars: 40 µm. (F–H) Box plots showing the density of ARG1^+^ cells (F), HIF1α^+^ cells (G), and double-positive ARG1^+^HIF1α^+^ cells (H) within the analyzed ROIs (8 to 10/group) using mIF imaging data. Representative results from 2 independent experiments are presented. *P* values were calculated using a 2-sided Wilcoxon rank-sum exact test. *P *<* 0.5; **P *<* 0.1; ***P *<* 0.001.

ST analyses of the inflammation-vascular axis, focusing on immune components infiltrating the vascular TME revealed the highest accumulation of neutrophils expressing colony-stimulating factor 3 receptor (*Csf3r*) and CD11b (*Itgam*) after OV/ACT combination treatment, compared to tumors treated with ACT or untreated controls (Fig. 4A, B). Infiltration of F4/80^+^ tumor-associated macrophages (TAMs) expressing *Adgre1*, and CD11c^+^ monocytes/dendritic cells (DCs) expressing *Itgax*, was more abundant after ACT, whereas frequencies of CAFs expressing fibroblast activation protein (*Fap*), B cells expressing *Cd19*, and NK cells expressing *Klrb1c* were comparable across all analyzed groups. Consistent with an influx of neutrophils/polymorphonuclear (PMN)–myeloid-derived suppressor cells (MDSCs) into the perivascular area, expression levels of chemokine receptors and chemokines, including *Cxcr2/4* and *Ccl2*, dominated the landscape after the OV-Fc/ACT combination, though their expression was reduced in animals treated with OV-CXCR4-A (Fig. S6A, B). The increased transcription of *Ccr5*, along with *Cxcl9*/*10* and *Ccl5* chemokines after the ACT monotherapy supported the higher infiltration of monocytes/DCs and TAMs. Aligned with evidence that PMN-MDSCs and anti-inflammatory TAMs are key determinants in establishing a T cell–excluded tumor phenotype,^28^ transcription levels of immunosuppressive markers were higher after ACT and OV-Fc/ACT treatments (Fig. 4C, D). This included the leukocyte-associated immunoglobulin-like receptor gene (*Lair1*), which inhibits immune cell activation upon binding to collagen and collagen domain-containing proteins,^29^ along with *Cd274* and *Pdcd1lg2*, arginase 1 (*Arg1*), nitric oxide synthase (*Nos2*), and *Fas*. The OV-Fc/ACT combination further augmented the immunosuppressive landscape of the perivascular stroma through PMN-MDSC–expressing genes such as *Chil3*, *Msr1*, *Arg2*, *C5ar1*, *Emilin2*, *Hif1a*, *Trem1*, *Clec4d*, and *Clec4e*, as well as elevated *S100a9* levels in mononuclear (M)-MDSCs.^30^ In contrast, transcription levels of most immunosuppressive molecules were downregulated after the OV-CXCR4-A/ACT combination (Fig. 4C, D).

Among the immunosuppressive genes specifically affected by different treatments in the TME, *Arg1* was highly expressed following ACT monotherapy, while hypoxia-inducible factor 1α (*Hif1a*) was predominant after OV-Fc/ACT combination treatment. To validate these findings, we examined the expression profile of ARG1 and HIF1α using mIF imaging in the same ROIs employed for the colocalization analysis of KRT8^+^ cancer cells, CD31^+^ vasculature, and COL1α1^+^ protein fibers. Consistent with the ST analysis, the highest number of ARG1-expressing tumor-infiltrating myeloid cells was observed after ACT monotherapy (Fig. 4E [upper panel], F). In contrast, HIF1α expression was predominant in myeloid cells following OV-Fc/ACT treatment, with some cells coexpressing both antigens (Fig. 4E [lower panel], G, and H, respectively). Notably, the numbers of cells expressing HIF1α and/or ARG1 were reduced to nearly background levels in OV-CXCR4-A/ACT–treated tumors.

Given that PMN-MDSCs suppressor cells, TAMs, and DCs share overlapping surface markers, we performed flow cytometric analysis of CD45^+^CD11b^+^ myeloid cells isolated from omental tumors of control and OV/ACT-treated SCID mice. As shown in Figure S7A and B, the greatest influx of *Itgam*-expressing myeloid cells occurred following OV-Fc/ACT combination treatment. The proportions of CD11b^+^Ly6G^hi^Ly6C^lo^ PMN-MDSCs were comparable between control and ACT-treated mice; however, their numbers increased >5-fold after OV-Fc/ACT treatment and were approximately 40% higher than those observed in OV-CXCR4-A/ACT–treated mice. In contrast, the highest accumulation of F4/80^+^ TAMs was observed following ACT treatment alone, whereas infiltration of CD24^+^Ly6C^−^ DCs increased similarly after both OV/ACT combination treatments. Additional analyses of omental tumors from MOVCAR 5009-bearing *TgMISIIR-TAg-Low* mice revealed a comparable composition of myeloid cell subsets across ACT and OV/ACT treatment groups (Fig. S7C), consistent with our previous observations of myeloid populations within the peritoneal TME of syngeneic ovarian cancer models.^10^^,^^12^

### Analyses of the activation profiles of TCR_TAG_ T cells within the perivascular TME and the tdLNs

**Figure 5.**
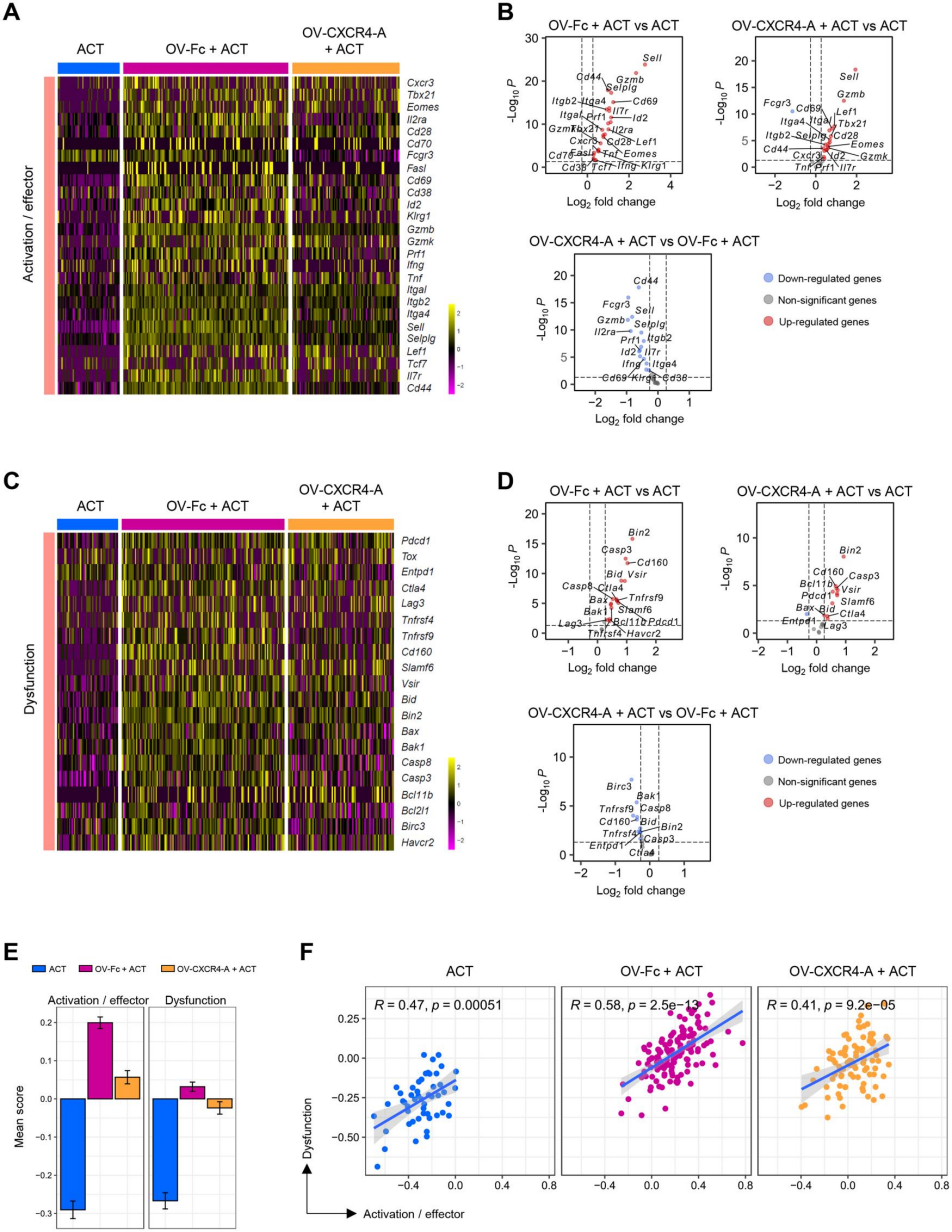
Analyses of differences in effector program acquisition of TCR_TAG_ T cells within the perivascular TME analyzed using spatial transcriptomics. (A–D) ST analysis of gene expression in TCR_TAG_ T cells infiltrating the perivascular TME of omental metastasis after ACT and OV treatments. (A) Expression heatmap showing activation/effector genes expressed by TCR_TAG_ T cells in different treatment groups. (B) Volcano plots of differentially expressed genes denoting an activated/effector phenotype of TCR_TAG_ T cells among the treatment groups. (C) Expression heatmap of genes denoting the dysfunctional phenotype of infiltrating TCR_TAG_ T cells across different treatment groups. (D) Volcano plots showing differentially expressed genes denoting a dysfunctional phenotype of TCR_TAG_ T cells among the treatment groups. In the volcano plots, each blue or red dot represents an individual gene with a Benjamini–Hochberg-adjusted *P* *<* 0.05, with red color denoting upregulated genes and blue color denoting downregulated genes. (E) Bar graphs showing treatment-mediated changes in gene set scores linked to activated/effector and dysfunctional states of tumor-infiltrating TCR_TAG_ T cells. Scores were calculated using the AddModuleScore function in the Seurat package, based on genes from (A) and (C), respectively. Data are presented as mean ± SD of the gene set score. (F) Correlations between activation/effector scores and dysfunction scores of TCR_TAG_ T cells across treatment groups. *n* = 50 spots for ACT-treated tumors, *n* = 134 spots for OV-Fc/ACT–treated tumors, *n* = 87 spots for OV-CXCR4/ACT–treated tumors.

We next examined how treatment-mediated changes in the perivascular TME affect the activation profile of tumor-infiltrating TCR_TAG_ T cells. Figure 5A shows that T cells transferred to untreated mice exhibited a low expression level of genes associated with stem-like (*Tcf7* and *Il7r*) and activation/effector (*Cd44*, *Cd69*, *Cxcr3*, *Prf1*, *Gzmb*, *Sell*) phenotypes, with only small increases in transcription of *Fcgr3*, *Ifng*, and *Cd38.* This contrasted with high activation profiles of TCR_TAG_ T cells induced by the OV/ACT combination treatments with either OV-Fc or OV-CXCR4-A (Fig. 5A, B), in line with the previous findings that co-stimulation signals are required for T cells to differentiate into effector cells.^31^ However, ACT/OV-Fc-induced inflammation contributed to higher transcription of genes associated with activation and effector differentiation of TCR_TAG_ T cells compared to those induced by the CXCR4-A–armed counterpart (Fig. 5A, B). These changes appeared more quantitative than qualitative, as both groups exhibited increased transcription of a similar set of genes relative to ACT monotherapy (Fig. 5B). The weak activation of TCR_TAG_ T cells was accompanied by reduced expression of genes associated with dysfunction (Fig. 5C), supporting the notion that a lack of co-stimulatory signals generally results in T-cell anergy linked to peripheral tolerance.^32^ The expression of some of these genes, including *Entpd1*, *Tnfrsf4*/*9*, *Cd160*, *Birc3*, and *Bak1* was higher in TCR_TAG_ T cells after OV-Fc/ACT than OV-CXCR4-A/ACT combination treatment (Fig. 5C, D). These differences were also reflected in the mean scores of the overall T-cell responses, which were lowest after ACT monotherapy, and highest and intermediate after OV-Fc/ACT and OV-CXCR4-A/ACT combinations, respectively (Fig. 5E). Correlations between the activation/effector and dysfunction gene set scores in TCR_TAG_ T cells were observed across all treatment groups (Fig. 5F), with the strongest association seen in OV-Fc/ACT–treated mice, which were characterized by impaired T-cell trafficking and reduced antitumor efficacy.

To verify the treatment-mediated changes in the activation and exhaustion profiles of the adoptively transferred TCR_TAG_ T cells, we analyzed the expression of the activation marker CD38^35^^,^^36^ and the co-inhibitory receptor LAG3^37^ on T cells in the omental tumors of MOVCAR 5009–bearing SCID mice. Flow cytometric analysis revealed that the tumor-infiltrating TCR_TAG_ T cells constituted less than 10% of CD45^+^ leukocytes (Fig. S8A), with the greatest increases after OV-CXCR4-A treatment. As shown in Fig. S8B, <5% of T cells expressed CD38 and LAG3 antigens after ACT treatment alone. Consistent with the ST findings, the proportions of TCR_TAG_ T cells expressing CD38^+^ or LAG3^+^ antigen increased approximately 4fold in OV-Fc/ACT–treated mice but remained lower following OV-CXCR-A/ACT treatment (*P* < 0.05). These results were corroborated by analysis of omental tumors from MOVCAR 5009-bearing *TgMISIIR-TAg-Low* mice, which exhibited modest increases in TCR_TAG_ T-cell infiltration and comparable activation and exhaustion marker expression profiles across ACT and OV/ACT treatment groups (Fig. S8C, D).

**Figure 6.**
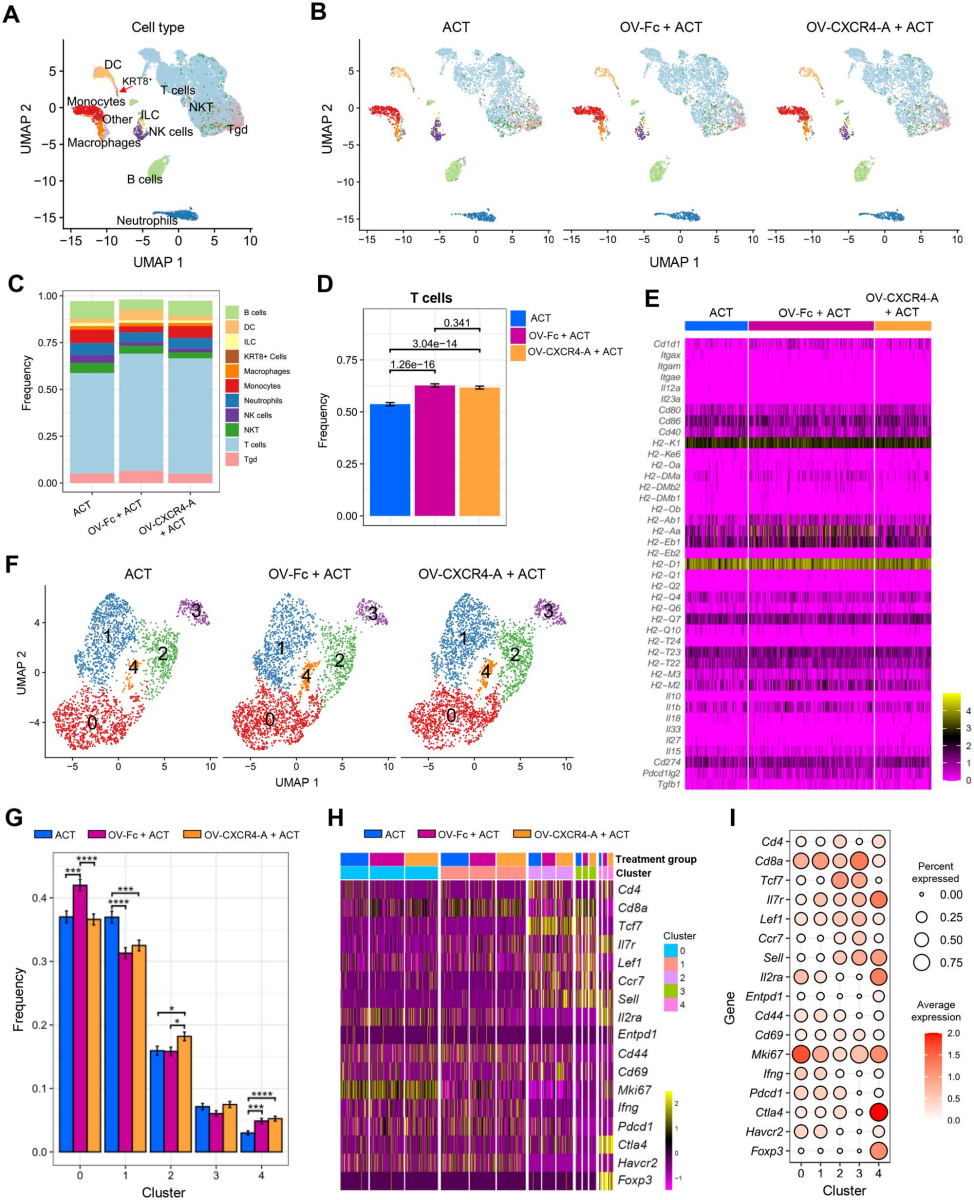
scRNA-seq analysis of TCR_TAG_ T cells in the tdLNs of MOVCAR 5009-bearing SCID mice after ACT and OV treatments. (A) UMAP visualization of cell populations in tdLNs analyzed 10 days after OV treatment. The red arrow indicates a population of *Krt8*-expressing tumor cells. (B) UMAP visualization of distinct cell populations in tdLNs from individual treatment groups with cell type colors corresponding to the respective cell type populations. (C) Bar plot representing the frequency of cell type populations in tdLNs after different treatments. Cell types in the data panel are highlighted by matching colors on the UMAP plots. (D) Frequency of TCR_TAG_ T-cell composition with significant changes among the indicated groups. Data are presented as mean ± SD, and the frequencies between groups were tested using a χ^2^ test. (E) Expression heatmap of DC activation markers. (F) UMAP visualization of reclustered TCR_TAG_ T-cell population comprising 5 (0 to 4) T-cell clusters in the tdLNs of SCID mice. (G) Bar plot depicting the frequencies of each T-cell cluster in tdLNs from different treatment groups. Data are presented as mean ± SD, and the frequencies between treatments were tested with a χ^2^ test. * *P* *<* 0.05, ***P *<* 0.001, ****P *<* 0.0001. (H) Heatmap showing the expression of selected genes within individual clusters across different treatment groups. (I) Dot plot illustrating the average expression and prevalence of genes among T-cell clusters. The size of each circle denotes the percentage of cells expressing the gene, and the color represents the average expression level in a cluster. *n* = 4 mice per group.

We next investigated whether the treatment-mediated changes in the trafficking profile of the adoptively transferred T cells occurred at the tumor site or were initiated in the tdLNs, consistent with molecular and cellular crosstalk between the tumor and the lymphoid tissues.^33^ Although SCID mice have been reported to have LNs that are less efficient in Ag presentation than their normal counterparts,^34^ they offer a distinct setting to explore the interaction between DCs and adoptively transferred T cells in the absence of potentially irrelevant endogenous immune cells.^35^ Enlarged inguinal LNs, visible only in the treated tumor-bearing mice, were resected at the time of tumor analysis and subjected to scRNA-seq. Figure 6A–D shows similar transcriptomic profiles of individual LN clusters across all treatment groups dominated by T cells, with approximately 20% increases in response to OV/ACT combinations compared to ACT monotherapy treatment. Less than 0.1% of cells in the tdLNs expressed the *Krt8* gene, suggesting early metastatic seeding; however, the tumor cell burden was insufficient to significantly alter the TME. The number of DCs was approximately 2-fold higher after the OV-Fc/ACT combination treatment (Fig. S9A; *P* < 0.0001), consistent with the highest activation level of tumor-infiltrating TCR_TAG_ T cells. Despite these quantitative differences and increases in the expression of genes encoding class II antigens *H2-Aa*, *H2-Ab1*, *H2-Eb1*, and programmed cell death-ligand 2 (*Pdcd1lg2*) (Fig. S9B), the transcriptional profile of key activation antigens on the DC populations was similar (Fig. 6E). This similarity was further reflected in the lack of heterogeneity within T-cell subsets identified through cluster analysis (Fig. 6F). T cells within the dominant clusters 0 and 1 were enriched after OV-Fc/ACT and ACT treatments, respectively (Fig. 6G). They expressed genes associated with activation (*Il2ra*, *Cd44*, *Cd69*, *Cd28*) and proliferation (*Mki67*), showed low expression of the co-inhibitory receptor *Havcr2*, and were negative for the expression of *Entpd1*, which is associated with exhaustion^36^ (Fig. 6H, I; Fig. S10). The majority of T cells in cluster 2, which increased after the OV-CXCR4-A/ACT combination, were stem-like (*Tcf7*^+^, *Pdcd1*^+^, and *Havcr2*^−^) with the potential to migrate to the tumor site, where they undergo differentiation into the effector state.^31^ In contrast, naïve cells (*Sell*^+^ and *Pdcd1*^−^) primarily made up cluster 3. Small numbers of CD4^+^ and CD8^+^ cells expressing *Foxp3*, *Il2ra*, and *Ctla4* were concentrated in cluster 4 with increased representation after OV/ACT treatments. Consistent with the transcriptomic study, flow cytometry analysis of TCR_TAG_ T cells isolated from the tdLNs at the time of scRNA-seq revealed a similar expression profile of CD62L, CD44, CD69, and PD1 across all treatment groups (Fig. S11A–D). Altogether, the presence of antigen-experienced T cells in the tdLNs of all treatment groups, with a stem-like phenotype and the ability to migrate into the tumor bed,^31^ suggested that the TME plays a central role in driving effector T-cell differentiation.

## Discussion

In this study, we combined the OV-based treatment approach with adoptively transferred TCR_TAG_ T cells to target TAG^+^ MOVCAR 5009 ovarian tumor, aiming to better understand how modulation of the perivascular niche influences antitumor T-cell trafficking and function in OC. We demonstrated that treatment with either the control or CXCR4-A–armed oncolytic vaccinia virus reduced MVD but had no significant effect on either the expression of PDGFRβ on the EC-associated pericytes or ICAM-1 on the vascular ECs unless used in combination with adoptively transferred TCR_TAG_ T cells. This occurs despite significant differences in survival and the tumor burden among the treatment groups, suggesting that activated T cells could drive upregulation of these molecules through the production of IFN-γ.^37^ We also observed profound alterations in the levels of angiogenic receptors and growth factors across treatment groups. These factors were most highly expressed in control mice and those receiving ACT alone, and lowest in the group treated with the OV-CXCR4-A/ACT combination. This pattern suggests a potential causal relationship between the reduced angiogenic signaling and the enhanced antitumor efficacy seen with the OV-CXCR4-A/ACT treatment, possibly mediated by the virus’s ability to suppress MVD and reprogram tumor-associated myeloid cells.

We propose that pretreatment with OV-Fc prior to ACT heightened activation of TCR_TAG_ T cells, which may have been counteracted by subsequent T-cell dysfunction, likely driven by an influx of immunosuppressive elements into the TME. These observations are consistent with our previous findings in syngeneic breast and ovarian cancer models, which showed increased intratumoral infiltration of MDSCs following treatment with control OV compared to the CXCR4-A–armed variant.^10^^,^^12^ Among antigens that were expressed in myeloid cells after OV-Fc but suppressed after OV-CXCR4-A treatment combination was HIF1α. Known for its multifactorial tumor-promoting effects, including angiogenesis, hypoxia, upregulation of several immune checkpoint proteins, as well as the induction of ARG1 in MDSCs,^38^ the high expression level of HIF1α could contribute to the dysfunction of TCR_TAG_ T cells and impair their trafficking. Additionally, increased transcription of some matrix metalloproteinases and lysyl oxidases, which are known for involvement in ECM crosslinking and remodeling,^39^ after treatment with OV-Fc/ACT combination (Fig. S12A, B), could also limit the migration of TCR_TAG_ T cells. In contrast, OV-CXCR4-A treatment, characterized by the secretion of CXCR4-A protein into perivascular tumor areas from virally infected tumor cells, reduced the immunosuppressive network in the TME.^8^^,^^10^^,^^12^ This modulated the level and quality of myeloid cell responses associated with the generation of functional TCR_TAG_ T cells. Altogether, these results further support the mutual interplay between the tumor vasculature and antitumor immunity^37^^,^^40^ and suggest improved vascular-immune crosstalk in tumor-bearing mice treated with the OV-CXCR4-A/ACT combination.

Our findings highlight the pivotal role of the magnitude and quality of co-stimulatory signals within the perivascular niche in regulating tumor Ag-specific T-cell differentiation and trafficking. Previous studies have shown that both co-stimulation and cytokines are critical for T-cell differentiation, as T cells that receive initial activation through MHC peptide interaction with the TCR and co-stimulation from various receptors without receiving a third signal from cytokines undergo poor proliferation and express lower levels of effector molecules.^31^ This aligns with the activation profile of TCR_TAG_ T cells delivered to untreated tumor-bearing mice. These results are in line with the 2-stage model of tumor-specific CD8^+^ T-cell activation, wherein initial T-cell priming in tdLNs is followed by differentiation of tumor-infiltrating stem-like CD8^+^ T cells into effector cells within the tumor upon receiving additional co-stimulatory signals.^31^ This implies that Ag-specific T cells primed by DCs in the tdLNs must have access to their tumor targets and encounter favorable conditions in the perivascular TME for developing effector function. This reasoning is also supported by similar numbers and activation states of T cells observed in the tdLNs following treatment with either OV-Fc or OV-CXCR4-A. It is also interesting that although OV-CXCR4-A leads to more effective tumor control, it induces lower expression of effector and cytotoxic molecules in T cells compared to OV-Fc. This apparent paradox may be explained by a simultaneous decrease in T-cell exhaustion markers, potentially enhancing T-cell functionality over time rather than recruiting more T cells to the perivascular niche than the control virus. Supporting this latter hypothesis, spatial analysis revealed that T cells in OV-Fc/ACT–treated mice accumulate more densely within 50 µm of the tumor vasculature, whereas T cells in OV-CXCR4-A–treated mice are more broadly distributed throughout the perivascular space, suggesting distinct migratory or retention patterns that may influence their antitumor efficacy.

Given the rapid acquisition of dysfunctional characteristics by tumor Ag-specific CD8^+^ T cells in tumor-bearing mice,^41^ further studies are necessary to delineate the molecular and cellular mechanisms driving effector T-cell differentiation during OV-induced ICD. Ongoing studies aim to comprehensively validate these correlative findings functionally by modulating myeloid populations, either through pharmacological inhibition or adoptive transfer, to determine the extent to which the immunosuppressive TME underlies the differing antitumor outcomes observed between OV-Fc and OV-CXCR4-A treatments. By dissecting the complex regulatory network driven by tumor-specific TCRs in the context of bystander T cells, which differ in their specificities, activation states, and effector function,^42^ more effective strategies can be developed to elucidate the mechanisms of nonspecific endogenous T-cell recruitment in the antitumor immune response. Of particular relevance to our research is the coexistence of cancer and infection, which may activate cancer-ignorant T cells through TCR-dependent infection responses, thereby contributing to antitumor immunity.^43^ Consequently, ICD represents a promising therapeutic approach in cancer immunotherapy, especially when combined with other modalities critical for controlling OC and other malignancies. The demonstrated antitumor synergy between OV-CXCR4-A and adoptively transferred tumoricidal TCR_TAG_ T cells complements our previous study, in which the same oncolytic virus in combination with doxorubicin increased ICD of drug-resistant OC cells, reversed the immunosuppressive TME, and controlled metastatic growth in syngeneic mice.^8^ In line with these and other preclinical studies, multiple ICD inducers have been shown to synergize with immunotherapeutic strategies in cancer patients, for example, anti-HER2 anthracycline-based antibody conjugates that potentiate PD-1 blockade in breast cancer.^44^ Several strategies have also targeted the limitations of ICD, including intratumoral injection of pattern recognition receptor agonists or recombinant type I IFNs^45^ as well as inhibition of endogenous suppressors of adaptive immunity elicited by ICD, such as CD47 blocking with specific monoclonal antibodies.^46^ Collectively, these observations underscore diverse strategies to enhance the immunogenicity of clinically “cold” tumors by combining ICD inducers with complementary treatment modalities, ultimately aiming to develop regimens with superior clinical efficacy.

## Supplementary Material

vlaf084_Supplementary_Data

## Data Availability

Data are available upon reasonable request. The raw data of scRNA-seq and Spatial Transcriptomics have been deposited in the database of Gene Expression Omnibus (GEO) available at ncbi.nlm.nih.gov/geo/under the accession numbers GSE274508 and GSE274509, respectively.
