# Supplementary material for: Consequences of the perivascular niche remodeling for tumoricidal T-cell trafficking into metastasis of ovarian cancer
Source: Immunohorizons. 2026 Feb 12;10(2):vlaf084. doi: 10.1093/immhor/vlaf084 (PMC12900543; doi:10.1093/immhor/vlaf084)
Supplement: vlaf084_Supplementary_Data [file vlaf084_supplementary_data.zip › Winkler et al. Supplementary material - IHORIZON-RA-2025-132.pdf]

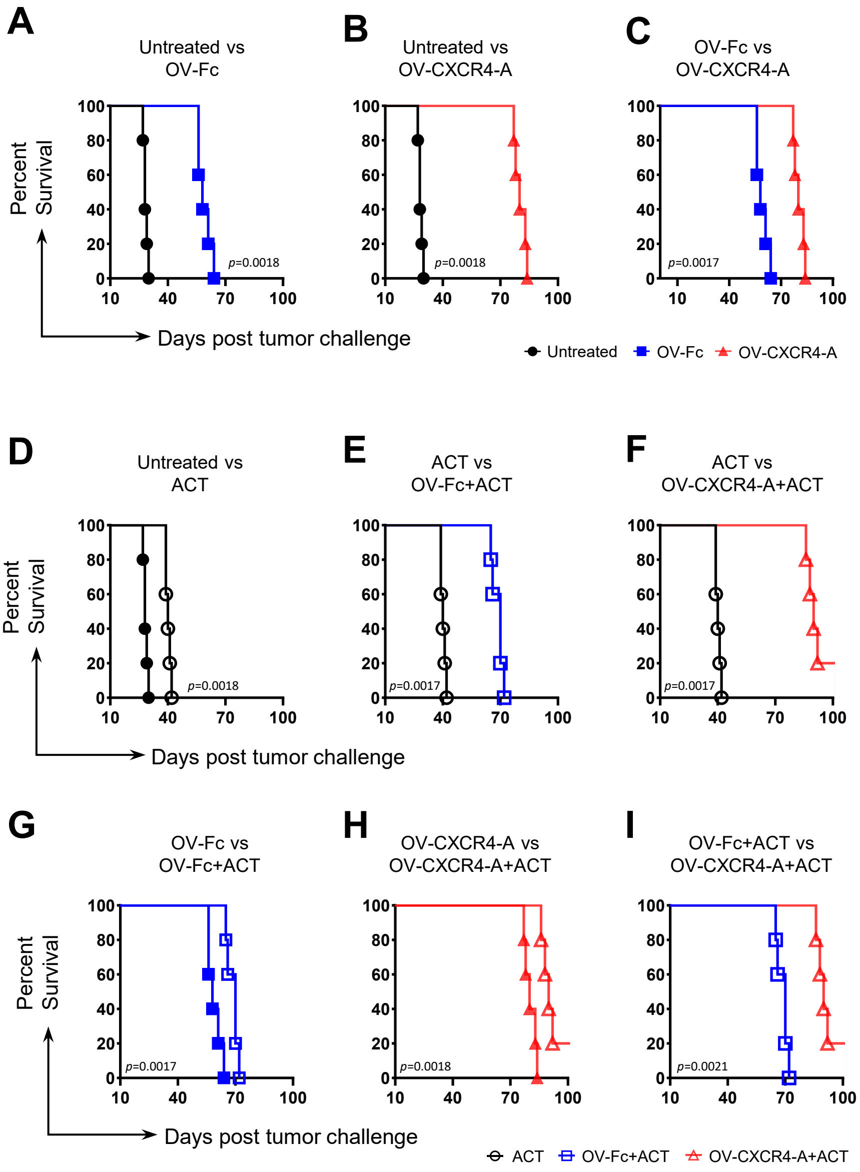

Figure S1. Effect of oncolytic virotherapy and adoptive cell transfer of TCR_{TAG} T cells on survival of tumor-bearing SCID mice.

Survival was defined as the time to euthanasia due to the development of abdominal distension. (A-I) Kaplan-Meier survival curves are shown. Statistical significance was determined using the log-rank (Mantel-Cox) test. *n* = 5 mice per group. Results are representative of three independent experiments.

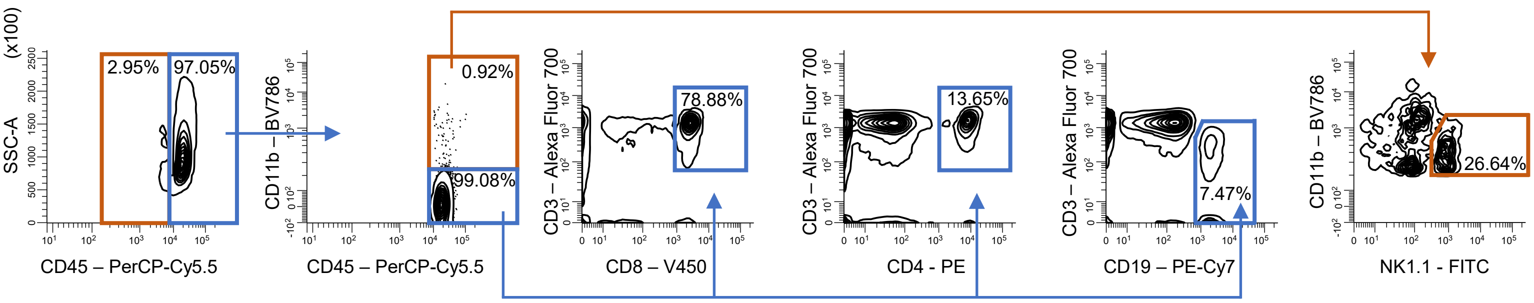

Figure S2. Gating strategy used to determine the phenotype of purified TCR_{TAG} T cells for adoptive transfer into tumor-bearing mice.

Representative flow cytometry plots show the phenotype of purified TCR_{TAG} T cells. A single-cell suspension obtained from the spleens of *B6.Cg-Tg(TcraY1,TcrbY1)416Tev/J* transgenic mice, was prepared using the Pan T Cell Isolation Kit II. Cells were stained with monoclonal antibodies specific for selected surface antigens and analyzed by flow cytometry. $CD45^{+}CD11b^{-}$ cells were gated and subsequently analyzed for CD3 and CD8 ($CD8^{+}$ T cells) or CD3 and CD4 ($CD4^{+}$ T cells) expression. $CD45^{+}CD11b^{-}CD3^{-}$ cells were examined for CD19 expression (B cells). Natural killer (NK) cells were identified as $CD45^{+}CD11b^{+}NK1.1^{+}$.

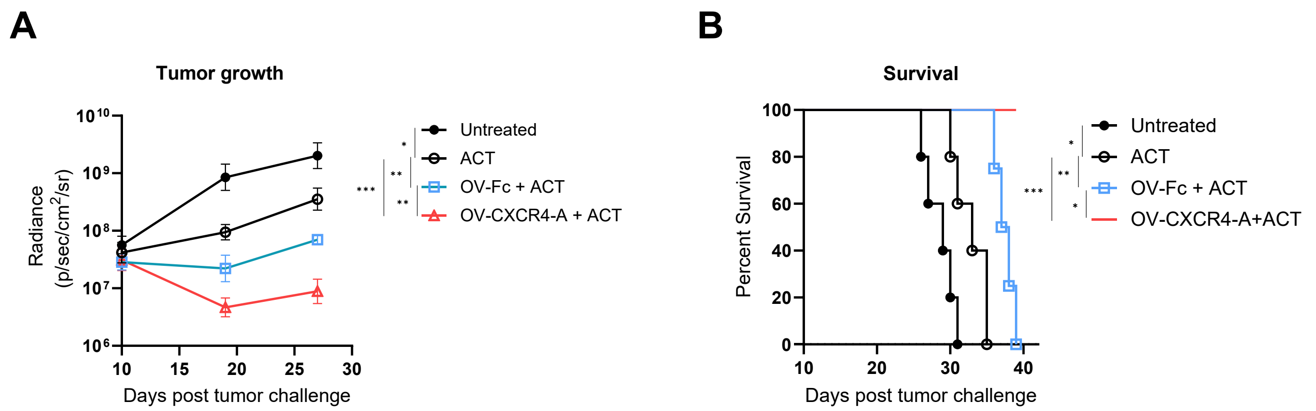

Figure S3. The effect of adoptive TCR_{TAG} T cell transfer and OV on MOVCAR 5009 tumor growth (A), and survival of *TgMISIIR-TAg-Low* mice (B).

(A) Tumor progression was monitored by bioluminescence imaging on days 10, 19, and 27 post-tumor challenge. Data are presented as mean ± SD. Statistical significance was determined using two-way ANOVA. *$p < 0.05$; **$p < 0.01$; ***$p < 0.001$. $n = 5$ mice per group. Results are representative of two independent experiments. (B) Kaplan–Meier survival curves showing the time to euthanasia following the development of abdominal distension. Statistical significance was assessed using the log-rank (Mantel-Cox) test. *$p < 0.05$; **$p < 0.01$; ***$p < 0.001$. $n = 5$ mice per group.

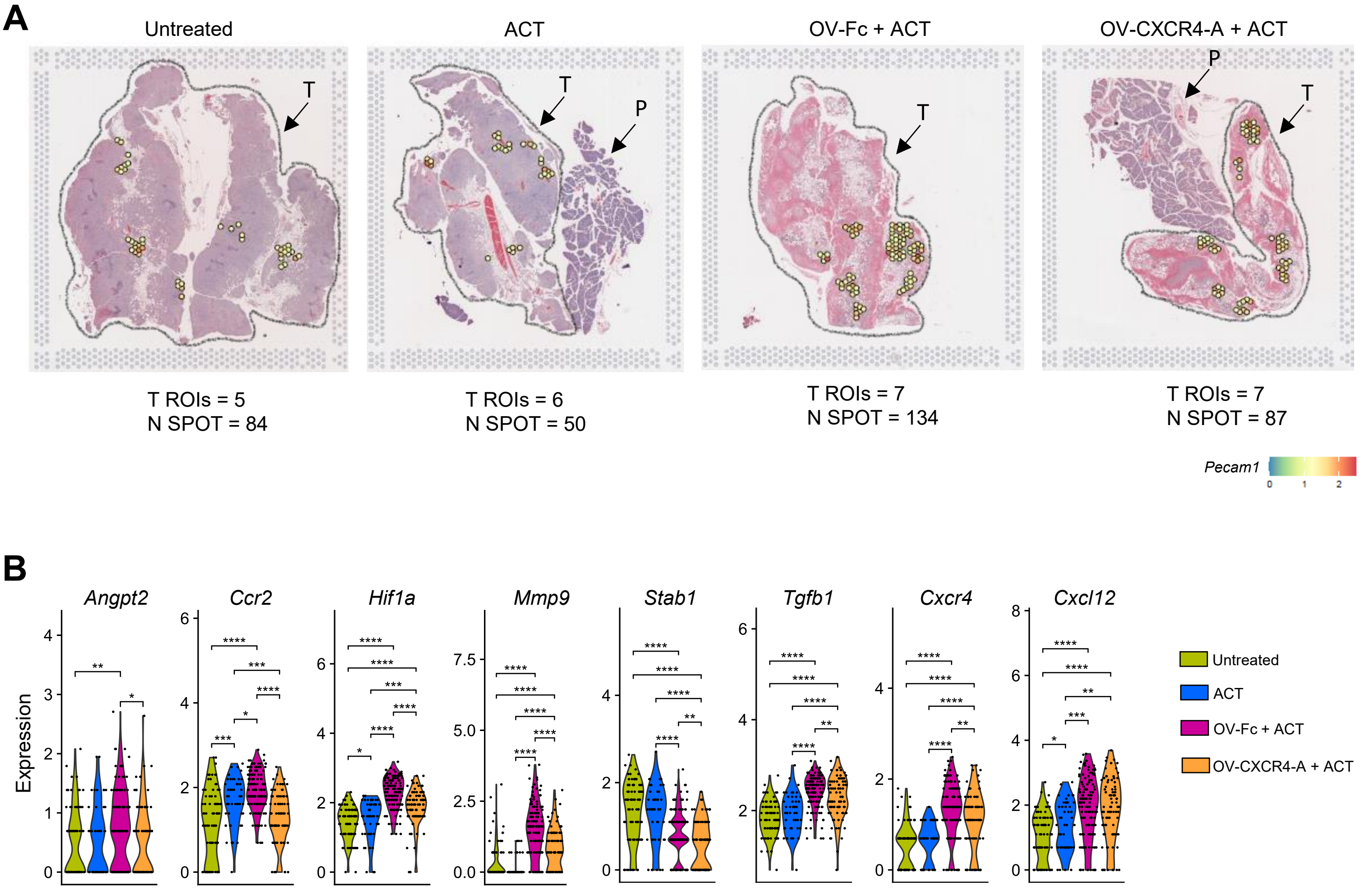

Figure S4. Spatial transcriptomic analysis of perivascular TME following monotherapy with ACT or the combination treatment with OV/ACT (A) and the effect of the treatment on the expression levels of genes encoding angiogenic receptors and growth factors (B).

(A) Formalin-fixed paraffin-embedded (FFPE) sections from omental tumors collected 10 days after virotherapy were analyzed by spatial transcriptomics. Tumor (T) and pancreas (P) regions are indicated. For each tumor section, five to seven regions of interest (ROIs) containing organized CD31-expressing tumor vasculature networks were selected. Within each ROI, 55 × 55 µm spots (represented as dots) were selected based on *Pecam1* (CD31) and *Cd3d* expression to investigate the perivascular niche. (B) Violin plots displaying treatment-mediated changes in the expression levels of genes encoding angiogenic receptors and growth factors. *P* values were calculated using a two-sided Wilcoxon rank sum exact test. *$p < 0.05$; **$p < 0.01$; ***$p < 0.001$; ****$p < 0.0001$.

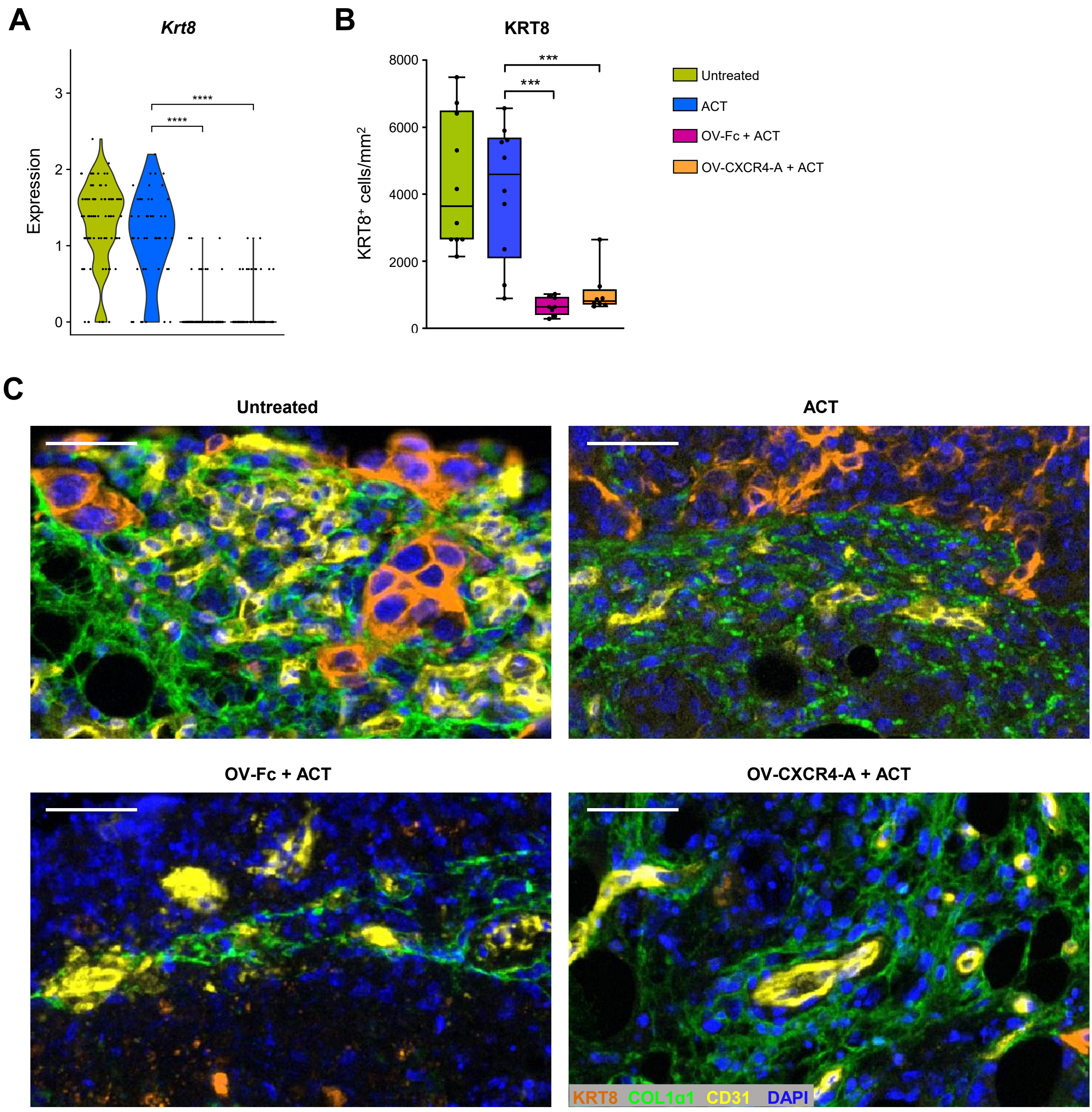

Figure S5. Treatment-mediated changes in KRT8 expression in ovarian cancer cells and COL1α1 fibers organization within perivascular sections of omental tumors.
(A) Violin plot of *Krt8* gene expression derived from Visium transcriptome data. n = 84 spots for untreated tumors, n = 50 spots for ACT-treated tumors, n = 134 spots for OV-Fc/ACT-treated tumors, n = 87 spots for OV-CXCR4/ACT-treated tumors. (B) Box plot of KRT8-positive cancer cells across 8-10 ROIs per group based on mIF imaging. P values were calculated using a two-sided Wilcoxon rank sum exact test. ***$p < 0.001$; ****$p < 0.0001$. (C) Panoramic views of representative ROIs illustrating treatment-mediated changes in the colocalization of CD31-expressing vascular ECs, KRT8-expressing tumor cells, and COL1α1 fibers within the perivascular TME of omental tumors. Representative results from two independent experiments are presented. DAPI was applied to visualize nuclei. CD31-Opal 480; KRT8-Opal 570; COL1α1-Opal 620. Scale bars: 40 μm.

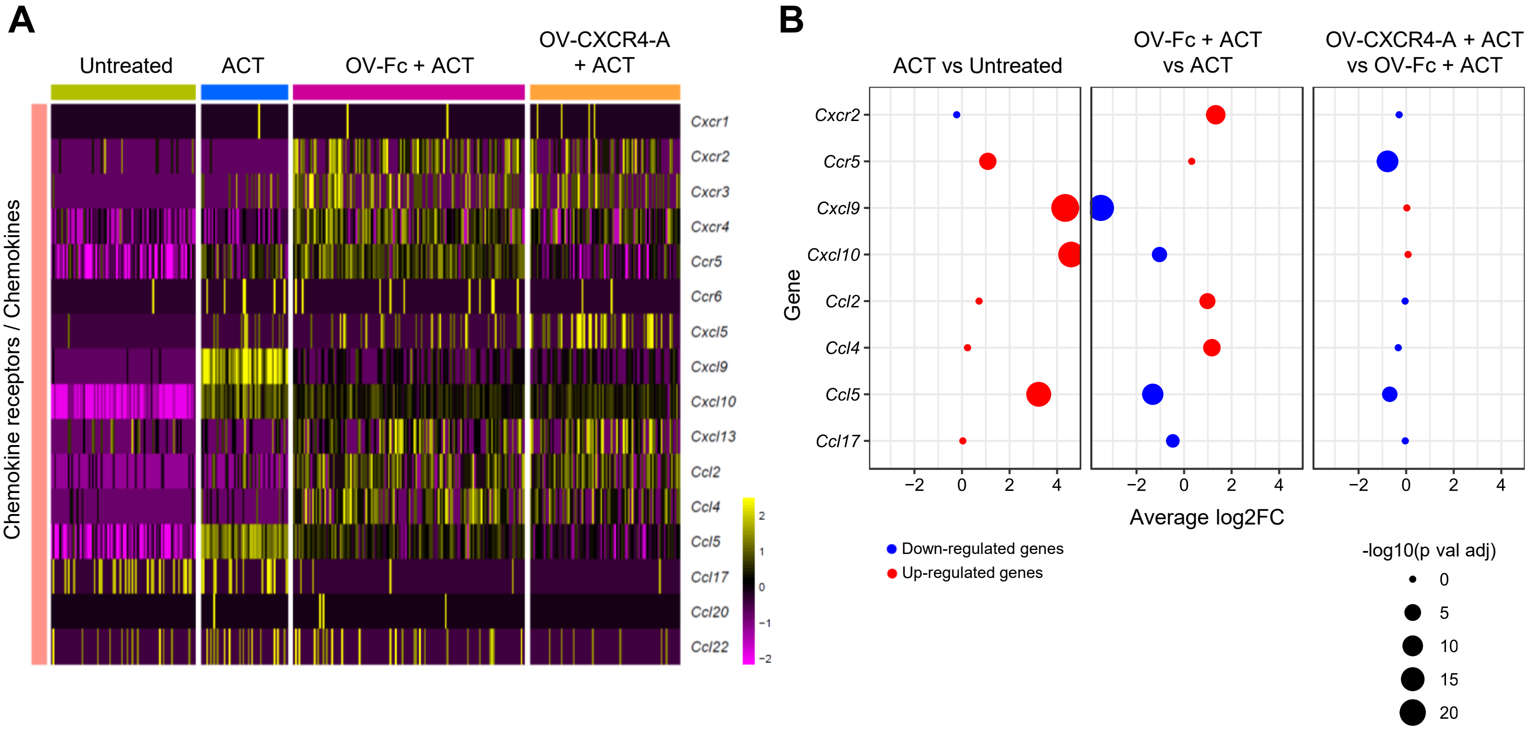

Figure S6. Treatment-induced changes in the expression of chemokine receptor and chemokine genes in the perivascular TME of omental tumors.

(A) Heatmap showing the expression profiles of genes encoding chemokine receptors and chemokines within the perivascular niche of omental tumors following OV and ACT treatments. (B) Dot plot illustrating differential expression of chemokine and chemokine receptor genes across treatment groups. The size of the dot represents a negative log10 adjusted *p* value, and the color indicates upregulation (red) or downregulation (blue) of genes. n = 84 spots for untreated tumors, n = 50 spots for ACT-treated tumors, n = 134 spots for OV-Fc/ACT-treated tumors, and n = 87 spots for OV-CXCR4/ACT-treated tumors.

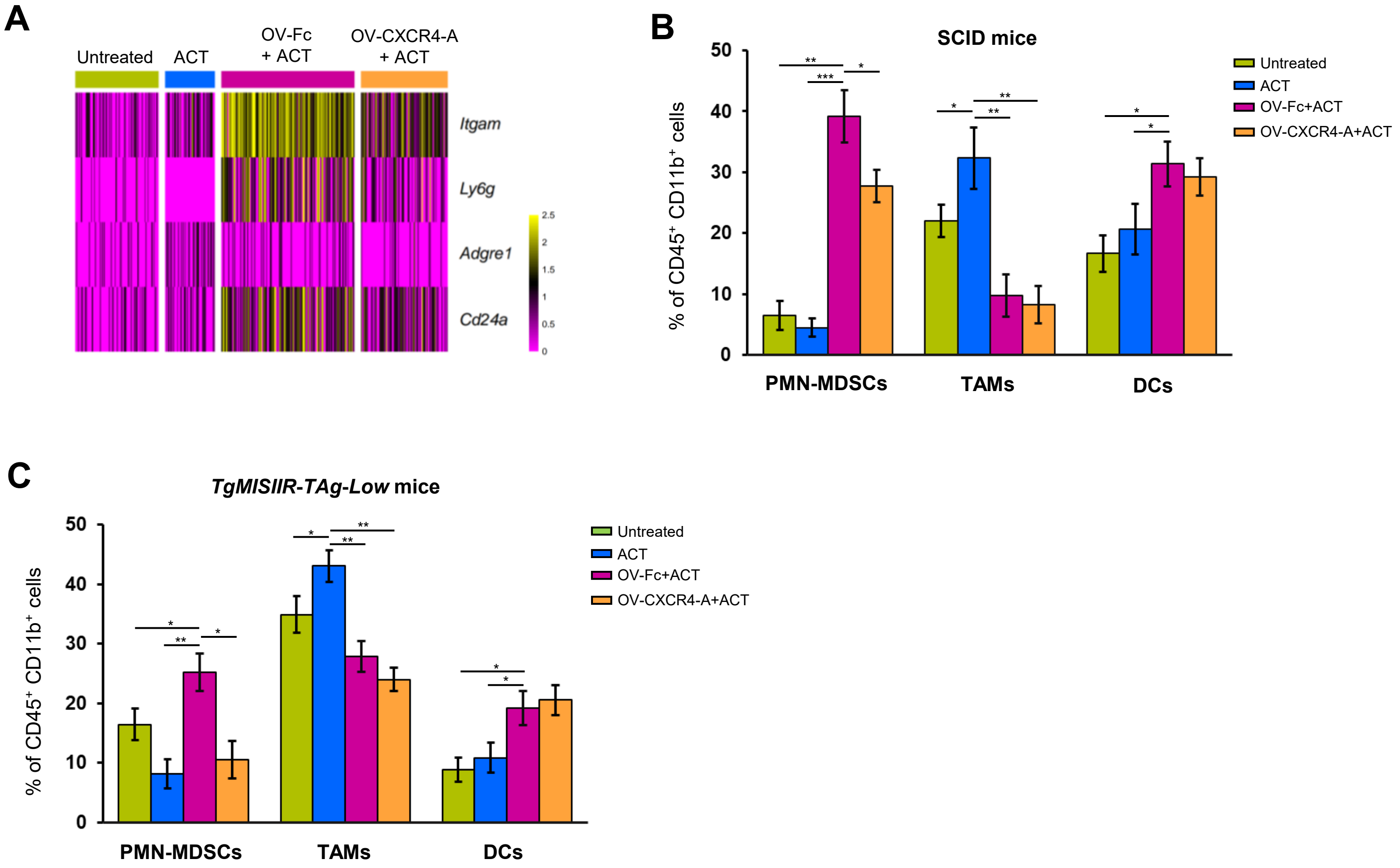

Figure S7. Heterogeneity of tumor-infiltrating myeloid populations in SCID and *TgMISIIR-TAg-Low* mice following ACT/OV treatments.

(A) Heatmap depicting the expression of myeloid markers: *Itgam* (encoding CD11b), *Ly6g* (Ly6G), *Adgre1* (F4/80), and *Cd24a* (CD24) within the perivascular niche of omental tumors in SCID mice. n = 84 spots for untreated tumors, n = 50 spots for ACT-treated tumors, n = 134 spots for OV-Fc/ACT-treated tumors, and n = 87 spots for OV-CXCR4/ACT-treated tumors. (B) Bar plot showing the proportion of PMN-MDSC, TAM, and DC populations among $CD45^+$ $CD11b^+$ cells in SCID mice. (C) Bar plot showing the proportion of PMN-MDSC, TAM, and DC populations among $CD45^+$ $CD11b^+$ cells in *TgMISIIR-TAg-Low* mice. Results are presented as the means ± SD of two independent experiments. n = 3 mice/group. Statistical analysis performed using the Wilcoxon rank sum test. *$p < 0.05$; ** $p < 0.01$; *** $p < 0.001$.

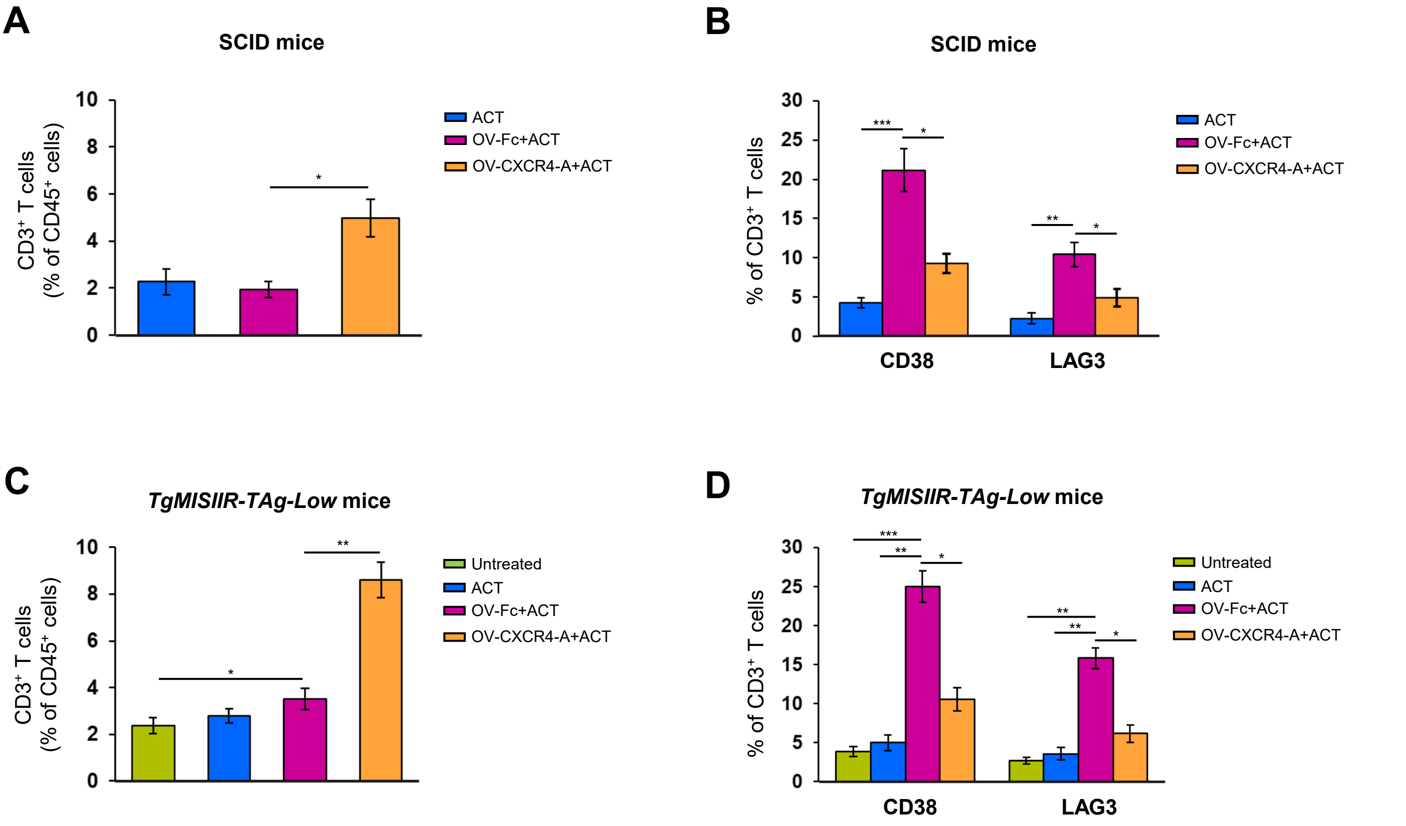

Figure S8. Phenotypic analysis of T cells in omental tumors of MOVCAR 5009-bearing SCID and *TgMISIIR-TAg-Low* mice following ACT and OV treatments.

(A-D) $CD3^+$ T cells in single-cell suspensions isolated from omental tumors of SCID (A and B) and *TgMISIIR-TAg-Low* (C and D) mice, ten days after treatment initiation, were analyzed by flow cytometry for the expression of T cell activation (CD38) and exhaustion (LAG3) markers. Percentages of $CD3^+$ T cells among $CD45^+$ leukocytes (A and C) and expression of CD38 and LAG3 on $CD3^+$ T cells (B and D) are shown. Data are presented as mean ± SD of two independent experiments. Statistical significance was assessed using the Wilcoxon rank sum test. $n = 3$ mice/group. * $p < 0.05$; ** $p < 0.01$; *** $p < 0.001$.

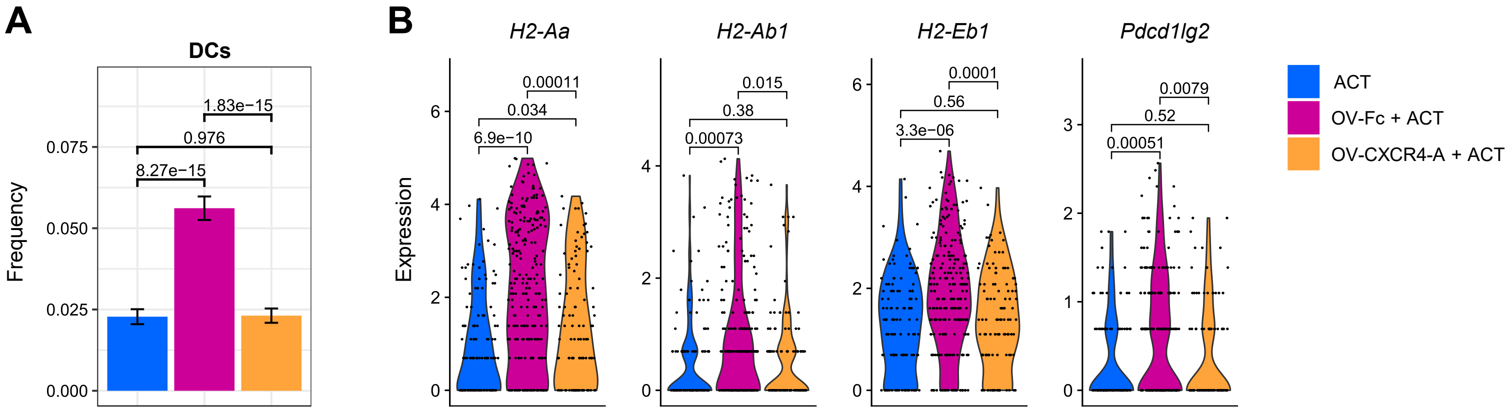

Figure S9. Analysis of treatment-mediated changes in the DC frequency and activation markers in the tdLNs.

(A) Frequencies of DCs showing significant differences between treatment groups were analyzed by scRNAseq. Data are presented as mean ± SD, and differences between groups were assessed using Chisquared test. (B) Violin plots showing differential expression of DC-associated across treatment groups. Statistical significance was calculated using the Wilcoxon rank-sum test. *n* = 4 mice/group.

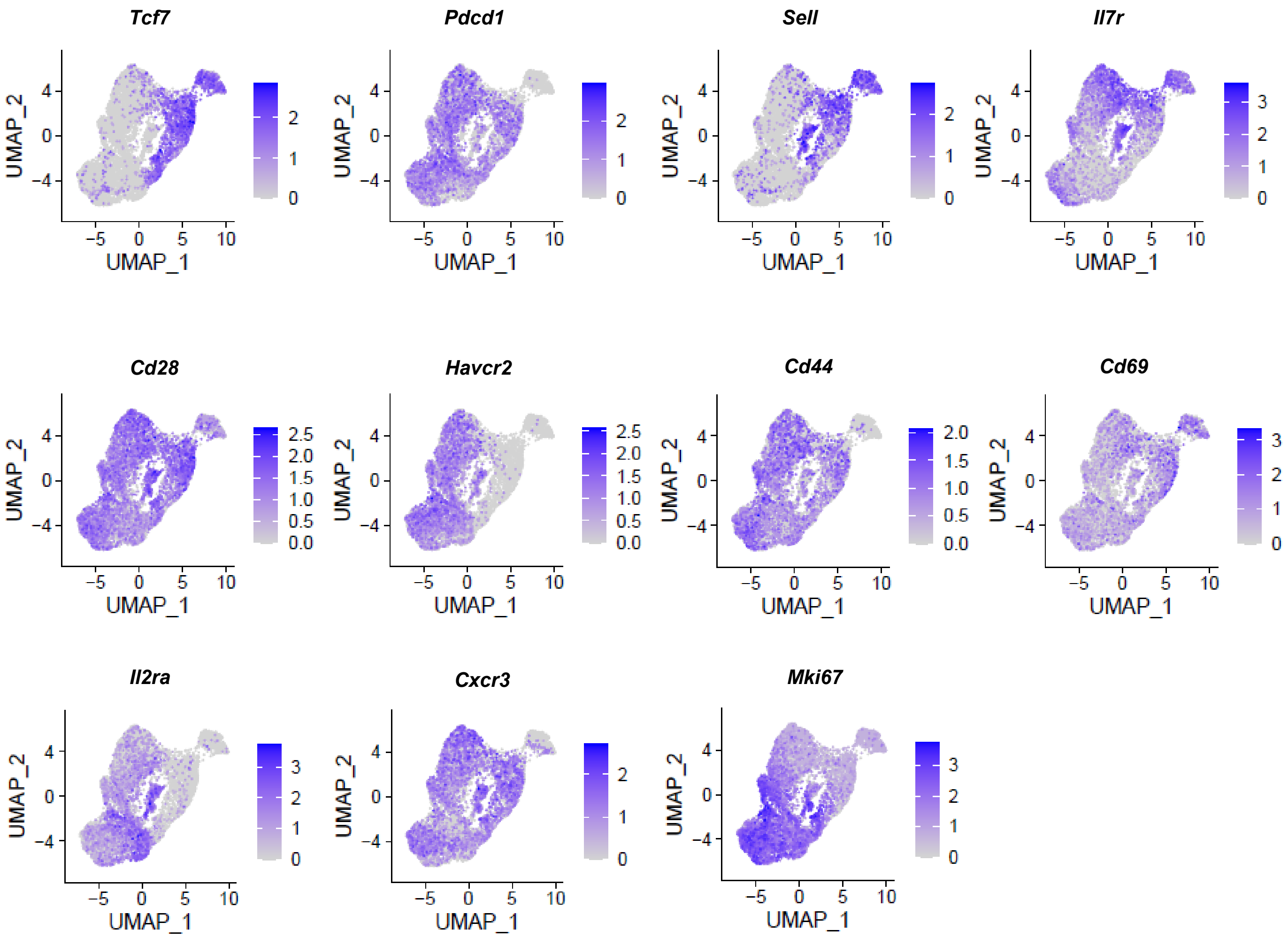

Figure S10. Treatment-mediated changes in gene expression profiles of TCR_{TAG} T cells in the tdLNs of tumor-bearing SCID mice.

FeaturePlots of re-clustered T-cell subsets in tdLNs showing expression of *Tcf7*, *Pdcd1*, *Sell*, *Il7r*, *Cd28*, *Havcr2*, *Cd44, Cd69, Il2ra, Cxcr3*, and *Mki67* are presented.

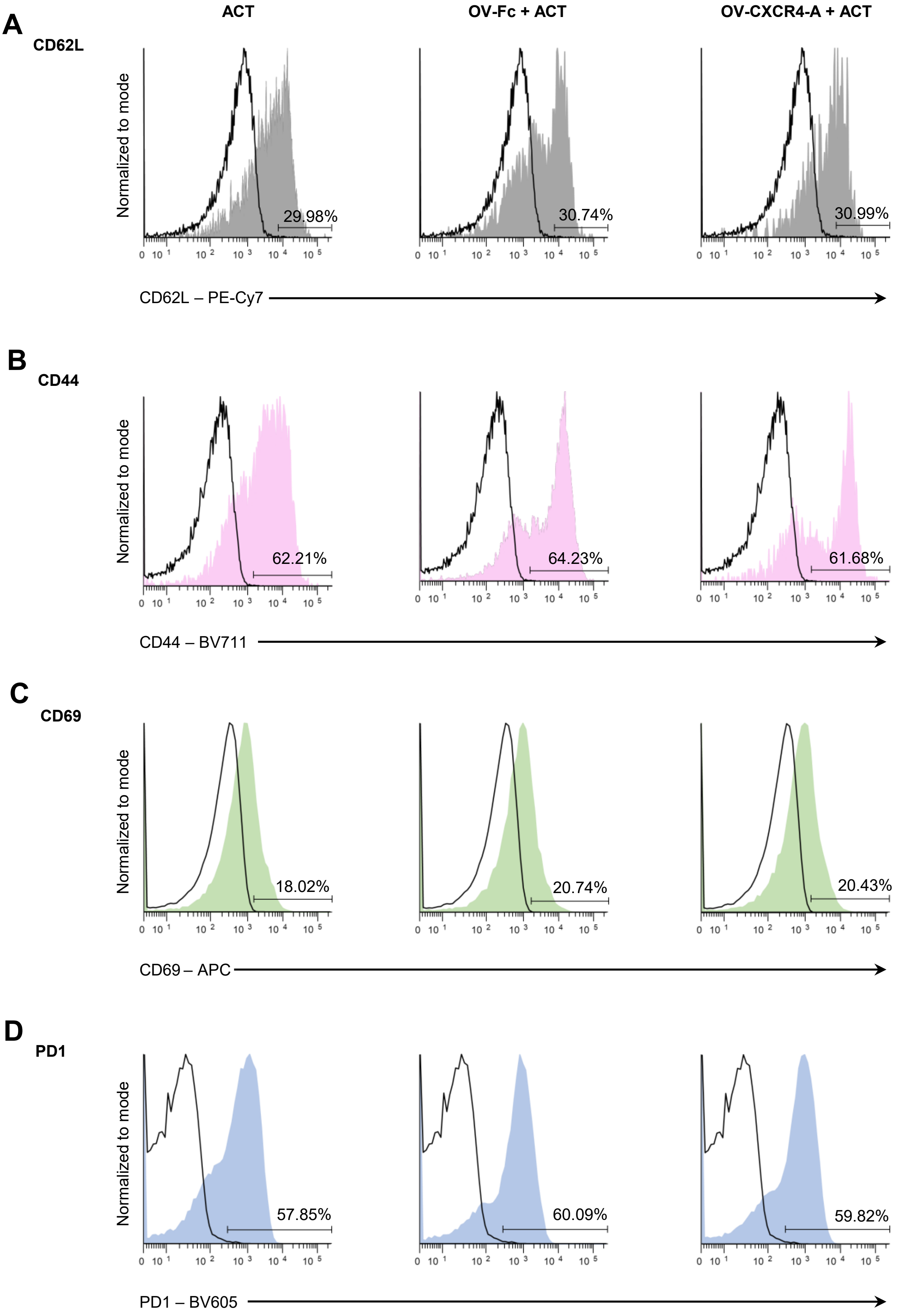

Figure S11. Flow cytometry analysis of TCR_{TAG} T cells in the tdLNs of SCID mice after ACT and OV treatments.

(A-D) Histograms showing expression of CD62L (A), CD44 (B), CD69 (C), and PD1 (D) on TCR_{TAG} T cells. Gates were set based on fluorescence minus one (FMO) controls (black lines). Percentages of T cells positive for each marker are indicated. *n* = 4 mice/group.

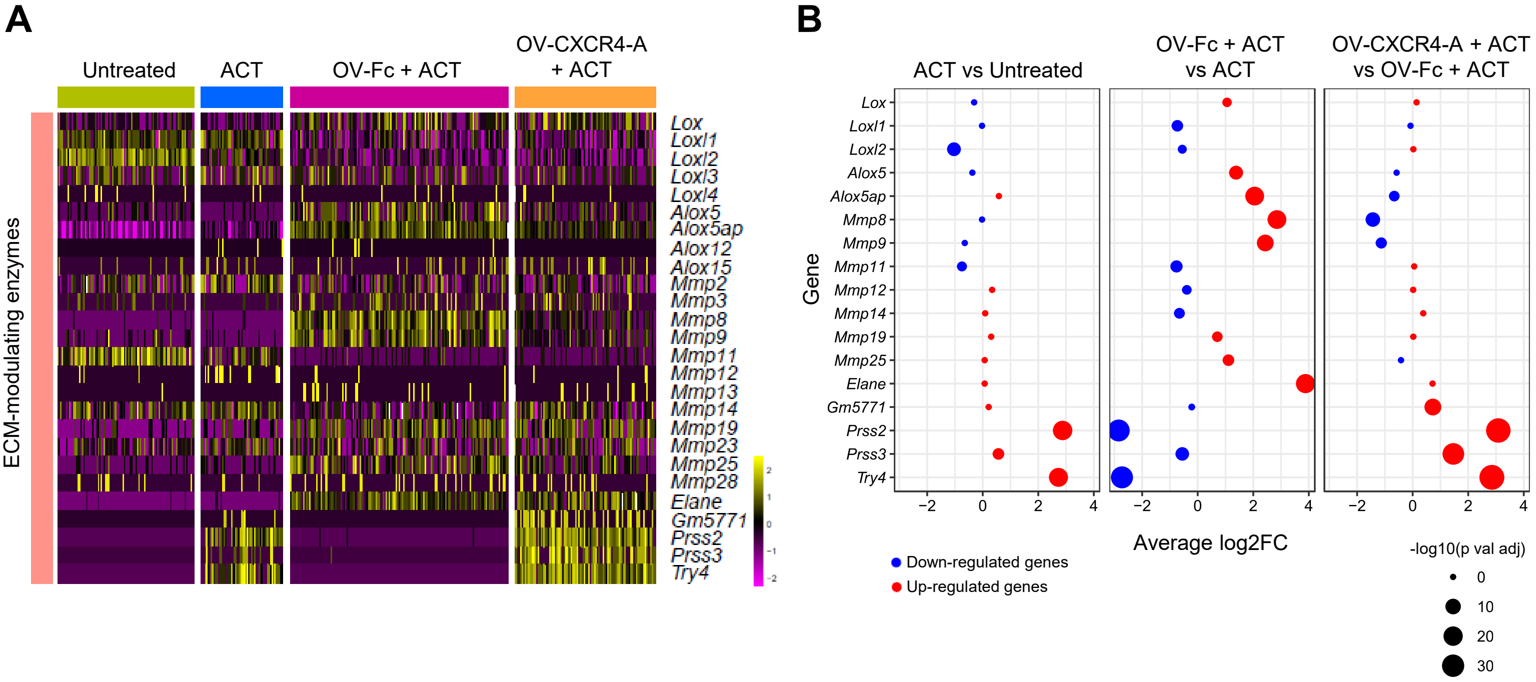

Figure S12. Treatment-mediated changes in the expression profile of ECM-modulating enzymes in the perivascular TME of omental tumors after ACT and OV treatments analyzed by spatial transcriptomics. (A) Heatmap showing the expression profile of ECM-modulating enzymes. (B) Dot plot illustrating differences in ECM-modulating enzyme expression levels among the treatment groups. The size of each dot represents a negative log10 Benjamini-Hochberg-adjusted p value, and color indicates upregulation (red) or downregulation (blue). n = 84 spots for untreated tumors, n = 50 spots for ACT-treated tumors, n = 134 spots for OV-Fc/ACT-treated tumors, and n = 87 spots for OV-CXCR4/ACT-treated tumors.

Table S1. Antibodies used in multispectral immunofluorescence (mIF) analysis.

Antibody	Clone	Catalog number	Source	Epitope retrieval	Dilution	Detection chemistry
**CD31, PDGFRβ panel**						
CD31	D8V9E	77699S	Cell Signaling Technology	ER1	1:100	Opal 570 at 1:100 for 10 mins
PDGFRβ	28E1	3169S	Cell Signaling Technology	ER2	1:100	Opal 520 at 1:150 for 20 mins
**CD31, ICAM-1, CD8 panel**						
CD31	D8V9E	77699S	Cell Signaling Technology	ER1	1:100	Opal 570 at 1:100 for 10 mins
ICAM-1	EPR16608	ab179707	Abcam	ER1	1:100	Opal 620 at 1:100 for 20 mins
CD8	EPR20305	ab209775	Abcam	ER1	1:100	Opal 690 at 1:150 for 10 mins
**CD31, ARG1, COL1α1, KRT8, HIF1α panel**						
CD31	D8V9E	77699S	Cell Signaling Technology	ER1	1:200	Opal 480 at 1:150 for 10 mins
HIF1α	E1V6A	48085S	Cell Signaling Technology	ER1	1:200	Opal 520 at 1:150 for 10 mins
KRT8	EP1628Y	ab53280	Abcam	ER1	1:550	Opal 570 at 1:150 for 10 mins
COL1α1	E8F4L	72026T	Cell Signaling Technology	ER2	1:200	Opal 620 at 1:150 for 10 mins
ARG1	D4E3M™	93668T	Cell Signaling Technology	ER2	1:400	Opal 690 at 1:150 for 10 mins

ER1 - citrate buffer pH 6, ER2 - Tris-EDTA buffer pH 9.

Table S2. Antibodies used in flow cytometry analyses.

Antibody	Clone	Catalog Number	Source	Dilution
CD45 – PerCP-Cy5.5	30-F11	550994	BD Biosciences	1:100
CD11b – BV786	M1/70	740861	BD Biosciences	1:200
CD3 – Alexa Fluor 700	17A2	561388	BD Biosciences	1:100
CD8 – V450	53-6.7	560469	BD Biosciences	1:100
CD4 – PE	GK1.5	553730	BD Biosciences	1:100
CD19 – PE-Cy7	1D3	561739	BD Biosciences	1:100
NK1.1 – FITC	PK136	108705	BioLegend	1:100
F4/80 – FITC	BM8	123107	BioLegend	1:100
Ly6G – PE	1A8	561104	BD Biosciences	1:100
Ly6C – PE-Cy7	AL-21	560593	BD Biosciences	1:200
CD24 – Alexa Fluor 700	M1/69	564237	BD Biosciences	1:100
CD38 – FITC	Ab90	558813	BD Biosciences	1:100
LAG3 – BV650	C9B7W	740560	BD Biosciences	1:100
CD62L – PE-Cy7	MEL-14	560516	BD Biosciences	1:100
CD44 – BV711	IM7	563971	BD Biosciences	1:100
CD69 – APC	H1.2F3	104513	BioLegend	1:200
PD1 – BV605	29F.1A12	135219	BioLegend	1:100
